# SQSTM-1/p62 potentiates HTLV-1 Tax-mediated NF-κB activation through its ubiquitin binding function

**DOI:** 10.1038/s41598-019-52408-x

**Published:** 2019-11-05

**Authors:** Aurélien Schwob, Elodie Teruel, Louise Dubuisson, Florence Lormières, Pauline Verlhac, Yakubu Princely Abudu, Janelle Gauthier, Marie Naoumenko, Fanny-Meï Cloarec-Ung, Mathias Faure, Terje Johansen, Hélène Dutartre, Renaud Mahieux, Chloé Journo

**Affiliations:** 1 International Center for Research in Infectiology, Retroviral Oncogenesis Laboratory, INSERM U1111 – Université Claude Bernard Lyon 1, CNRS, UMR5308, École Normale Supérieure de Lyon, Université Lyon, Lyon, France; 2 Equipe labellisée “Fondation pour la Recherche Médicale”, Paris, France; 3 International Center for Research in Infectiology, Autophagy, Infection, Immunity Laboratory, INSERM U1111 – Université Claude Bernard Lyon 1, CNRS, UMR5308, École Normale Supérieure de Lyon, Université Lyon, Lyon, France; 4 Department of Biomedical Sciences of Cells and Systems, Section Molecular Cell Biology, University Medical Center Groningen, Groningen, The Netherlands; 5 Molecular Cancer Research Group, Institute of Medical Biology, University of Tromsø - The Arctic University of Norway, Tromsø, Norway; 6 École Normale Supérieure Paris-Saclay, Université Paris-Saclay, Cachan, France

**Keywords:** Leukaemia, HTLV, Cell signalling, Ubiquitylation, Tumour virus infections

## Abstract

The NF-κB pathway is constitutively activated in adult T cell leukemia, an aggressive malignancy caused by Human T Leukemia Virus type 1 (HTLV-1). The viral oncoprotein Tax triggers this constitutive activation by interacting with the ubiquitin-rich IKK complex. We previously demonstrated that Optineurin and TAX1BP1, two members of the ubiquitin-binding, Sequestosome-1 (SQSTM-1/p62)-like selective autophagy receptor family, are involved in Tax-mediated NF-κB signaling. Here, using a proximity-dependent biotinylation approach (BioID), we identify p62 as a new candidate partner of Tax and confirm the interaction in infected T cells. We then demonstrate that *p62* knock-out in MEF cells as well as *p62* knock-down in HEK293T cells significantly reduces Tax-mediated NF-κB activity. We further show that although *p62* knock-down does not alter NF-κB activation in Jurkat T cells nor in infected T cells, *p62* does potentiate Tax-mediated NF-κB activity upon over-expression in Jurkat T cells. We next show that p62 associates with the Tax/IKK signalosome in cells, and identify the 170–206 domain of p62 as sufficient for the direct, ubiquitin-independent interaction with Tax. However, we observe that this domain is dispensable for modulating Tax activity in cells, and functional analysis of p62 mutants indicates that p62 could potentiate Tax activity in cells by facilitating the association of ubiquitin chains with the Tax/IKK signalosome. Altogether, our results identify p62 as a new ubiquitin-dependent modulator of Tax activity on NF-κB, further highlighting the importance of ubiquitin in the signaling activity of the viral Tax oncoprotein.

## Introduction

Human T cell Leukemia Virus type 1 (HTLV-1) is a member of the *Retroviridae* family and of the *Deltaretrovirus* genus^1,2^. It infects at least 5 to 10 million people worldwide, notably in several endemic regions such as Japan, Sub-Saharan Africa, the Caribbean, Brazil and a part of Eastern Europe^3,4^. HTLV-1 is the etiologic agent of Adult T cell Leukemia (ATL) and of a set of inflammatory diseases including Tropical Spastic Paraparesis/HTLV-Associated Myelopathy (HAM/TSP)^5^. At the cellular level, HTLV-1 induces the constitutive activation of the NF-κB signaling pathway in infected T cells. This drives both cell transformation and inflammation^6,7^. The viral transactivator Tax promotes constitutive activation of both the canonical and non-canonical NF-κB pathways^8^.

In non-infected T cells, the canonical NF-κB pathway is activated downstream of several receptors, such as Toll-Like Receptors (TLR), Tumor Necrosis Factor Receptors (TNFR) and the T Cell Receptor (TCR). Regardless of the nature of the receptor, its engagement results in the recruitment of the IκB kinase (IKK) complex by K63-linked and linear M1-linked polyubiquitin chains borne by signaling intermediates, such as TRAF6, RIP1 or MALT1, or by unanchored polyubiquitin chains^9^. The IKK complex activation then promotes the IκBα inhibitor phosphorylation, followed by its ubiquitination and proteasomal degradation, allowing NF-κB nuclear translocation and target gene transactivation.

HTLV-1 Tax has been shown to recruit the IKKγ regulatory subunit of the IKK complex^10–12^ via direct interaction strengthened by Tax-conjugated K63-polyubiquitin chains^13–19^, leading to IκBα degradation and NF-κB activation^20^. In addition, recent studies also suggested that Tax could enhance synthesis of unanchored polyubiquitin chains by RNF8^21^, and of hybrid K63- and M1-linked polyubiquitin chains by LUBAC^22^. Tax could thus trigger IKK activation through indirect, ubiquitin-dependent interactions, by organizing an active macromolecular IKK signalosome. On the other hand, it was also suggested that Tax acts as an E3-ubiquitin ligase that directly catalyzes synthesis of unanchored polyubiquitin chains, although these results are still debated^23^.

The Tax/IKK signalosome has been described as a cytoplasmic complex associated with the centrosome and the Golgi^14,16,19^ that assembles mainly on lipid rafts^24^ by a mechanism that relies on the membrane-associated CADM1 protein^25^. In a previous work, we identified both Optineurin (OPTN) and Tax1-Binding Protein 1 (TAX1BP1) as crucial cellular partners involved in Tax-dependent NF-κB activation^26^. More specifically, OPTN was shown to interact with Tax in Golgi-associated structures and to enhance its K63-polyubiquitination in a TAX1BP1-dependent manner. OPTN and TAX1BP1 association with the Tax/IKK signalosome on lipid raft-enriched membranes in infected cell lysates was further confirmed by other investigators^25^. Independently, Shembade *et al*. demonstrated that Tax interaction with TAX1BP1 impaired the assembly of the inhibitory TAX1BP1/A20 complex, further contributing to constitutive NF-κB activation^27^.

Both OPTN and TAX1BP1 have recently been identified as members of the Sequestosome-1 (SQSTM-1/p62)-like selective autophagy receptor (SLR) family^28–31^, which function as selective macroautophagy receptors^32^. Interestingly, in addition to its function as a selective autophagic receptor, p62 has also been shown to potentiate NF-κB signaling downstream of TNFα and IL-1 stimulation by interacting with PKCζ, RIP1, TRAF6 and IKKγ, and enhancing TRAF6 E3 ubiquitin ligase activity towards TRAF6 itself and towards IKKγ^33–37^. p62 was further shown to interact with MEKK3 and to allow MEKK3 association with TRAF6, thereby allowing MEKK3-induced activation of NF-κB^38^. p62 also potentiates TRAF6-dependent NF-κB signaling downstream of CD40 activation in macrophages^39^. Upon TCR stimulation in T cells, p62 was identified as an essential scaffold that mediates clustering of the MALT1-BCL10-TRAF6 signalosome upstream of IKK activation^40^. Taken together, these findings suggest a crucial scaffolding hub function for p62 in NF-κB signaling. Herein, we identify p62 as a new partner of Tax and demonstrate that in addition to OPTN and TAX1BP1, p62 also potentiates Tax-mediated NF-κB signaling. We further show that p62 associates with the Tax/IKK signalosome in infected cells. Data obtained using p62 mutants suggest that p62 could potentiate Tax activity by binding to ubiquitin chains. Altogether, our results identify p62 as a new modulator of Tax activity on NF-κB and support a ubiquitin-dependent scaffolding role for p62 in this process.

## Results

### Proximity-dependent biotinylation by BirA*-Tax fusion protein identifies p62 as a new partner of Tax

**Figure 1:**
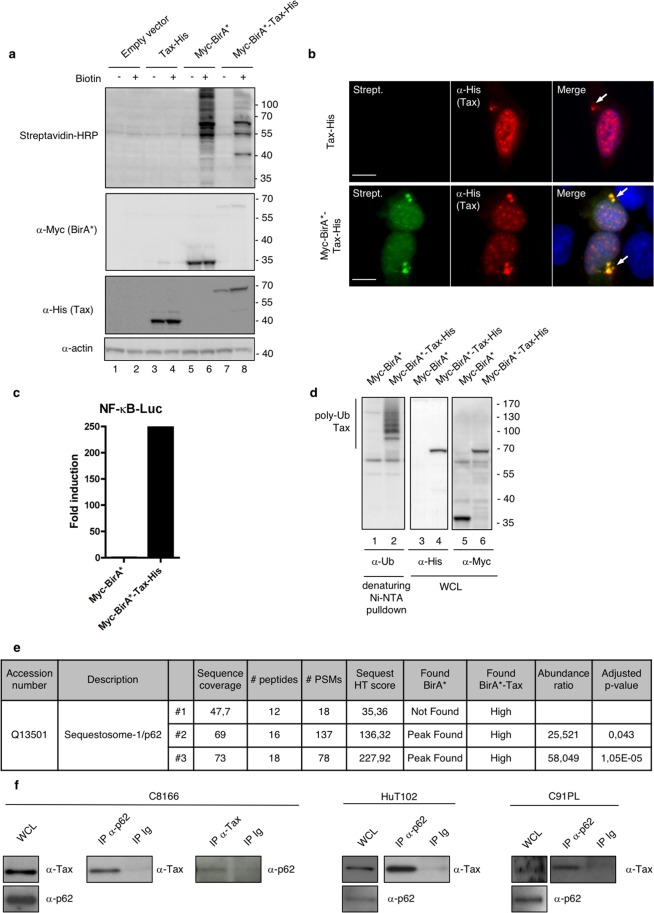
Functional validation of the BirA*-Tax fusion protein and identification of p62 as a new candidate partner of Tax. (**a**) Lysates from HEK293T cells transfected with the indicated plasmids for 24 h and then treated overnight with biotin or left untreated were analyzed by western blot. (**b**) U2OS cells transiently expressing Tax-His or Myc-BirA*-Tax-His and treated overnight with biotin were analyzed by epifluorescence microscopy after staining with Streptavidin (Strept., green) and His-specific antibodies (red). Nuclei were counterstained with DAPI (blue). Representative images are shown. Scale bar = 10 μm. The arrows indicate perinuclear accumulation of Tax reminiscent of the Tax/IKK signalosome. (**c**) HEK293T cells were transfected with Myc-BirA* or Myc-BirA*-Tax-His, together with an NF-κB-luc construct. Luciferase activity was measured and normalized over the “Myc-BirA*” condition. The graph shows the result from a representative experiment. (**d**) Lysates from HEK293T cells transiently expressing Myc-BirA* or Myc-BirA*-Tax-His were submitted to a His-specific Ni-NTA pulldown in denaturing conditions before western blot analyses. WCL, whole cell lysate. (**e**) SQSTM-1/p62 is a BirA*-Tax-specific biotinylated protein identified by mass spectrometry. (**f**) Lysates from HTLV-1 chronically infected cells (C8166, HuT102 or C91PL cells) were immunoprecipitated with a p62-specific or Tax-specific antibody, or with control Ig (IP Ig). Samples were then analyzed by western blot. WCL, whole cell lysates. Full-length blots are presented in Supplementary Fig. S4.

In order to identify new cellular partners of Tax involved in NF-κB signaling, we took advantage of BioID, a recently developed proximity-dependent labelling approach^41^. In this procedure, the protein of interest (here Tax) is fused to a biotin ligase domain modified from an *E. coli* enzyme (BirA*). Expression of this fusion protein in the presence of biotin allows proximity-dependent labelling of partners in a 10nm-radius. Biotinylated partners are then purified and analyzed by mass spectrometry. We first verified that the BirA*-Tax fusion protein was able to induce biotinylation (Fig. 1a). Of note, BirA*-Tax displayed the expected subcellular localization previously described for Tax, with nuclear speckles as well as a perinuclear accumulation of Tax reminiscent of the Tax/IKK signalosome associated with the Golgi apparatus^14^ (Fig. 1b, see arrows). BirA*-Tax-mediated biotinylation depended on proximity, as shown by the colocalization of BirA*-Tax and Streptavidin-stained biotinylated protein (Fig. 1b). Using a NF-κB-dependent luciferase reporter assay, we then verified that the BirA*-Tax fusion protein conserved its ability to activate the NF-κB pathway (Fig. 1c). The BirA*-Tax fusion protein conserved its ability to undergo polyubiquitination, a feature required for NF-κB signaling^13–19^, as shown by its purification by Ni-NTA pulldown under denaturing conditions followed by ubiquitin-specific western blotting (Fig. 1d). These control experiments indicate that the BirA*-fused Tax construct is appropriate for identifying cellular partners involved in NF-κB signaling.

We then purified the biotinylated proteins from BirA*-Tax-expressing cells and subjected them to mass spectrometry identification. As a control, biotinylated proteins from BirA*-only expressing cells were also analyzed. One of the top-ranked BirA*-Tax-specific protein identified in the three independent replicas was identified as Sequestosome-1 (SQSTM-1/p62, Fig. 1e), indicating that p62 could be a proximity partner of Tax.

We then verified whether Tax and p62 could interact in infected T cells. Co-immunoprecipitation assays were performed in HTLV-1 chronically-infected C8166, HuT102 and C91PL cells (Fig. 1f). Endogenous Tax and p62 co-immunoprecipitated in all these cell lines, confirming that p62 is indeed a partner of Tax.

### p62 potentiates Tax-dependent NF-κB activation

**Figure 2:**
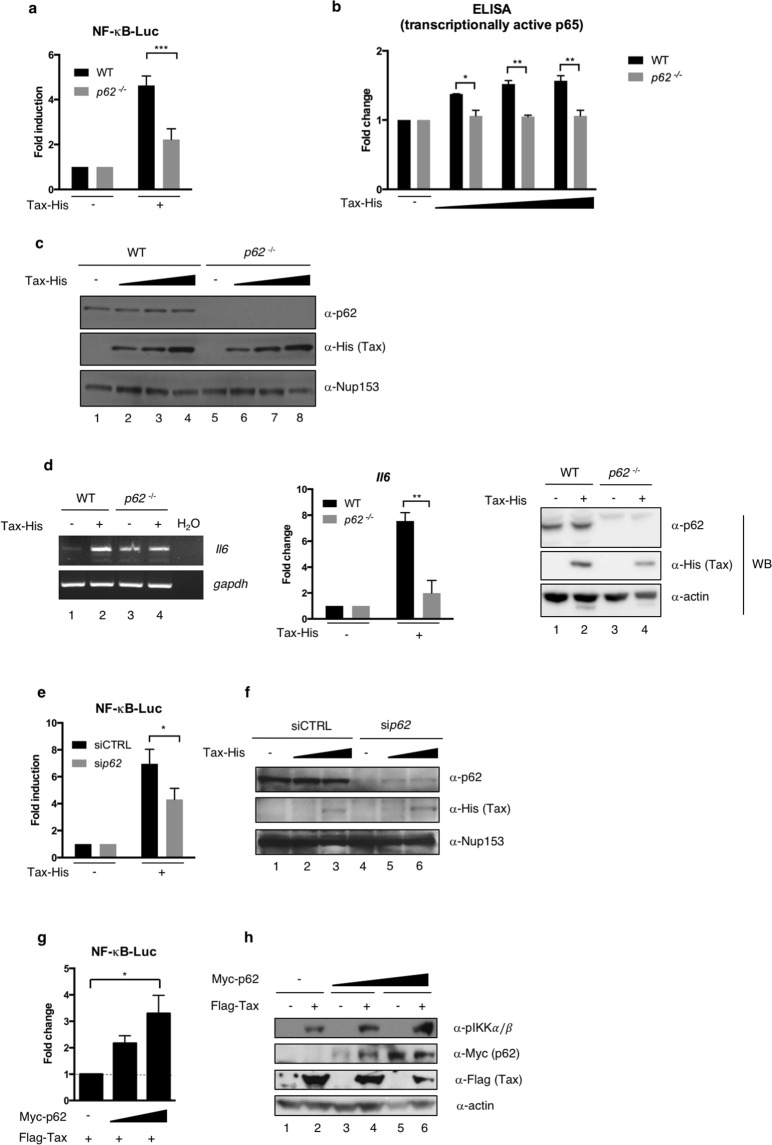
p62 potentiates Tax-dependent NF-κB activation upstream of IKK activation. (**a**) Wild type (WT) and *p62*^−/−^ MEF cells were transfected with Tax-His, together with an NF-κB-luc construct. Luciferase activity was measured and normalized over the corresponding Tax-negative condition. The graph shows results from at least 3 independent experiments. (**b**) WT and *p62*^−/−^ MEF cells were transfected with Tax-His. After fractionation of cell nuclei, transcriptionally active p65 was quantified by ELISA. (**c**) Lysates from WT and *p62*^−/−^ MEF cells transfected with Tax-His were analyzed by western blot. (**d**) WT and *p62*^−/−^ MEF cells were transfected with Tax-His. After RNA extraction and conversion to cDNAs, *Il6* and *gapdh* cDNAs were amplified by PCR (left panel). The normalized *Il6* signal intensities were calculated and are shown relative to the corresponding Tax-negative conditions on the graph (results from 2 independent experiments). Cell lysates were also analyzed by western blot (right panel). (**e**) HEK293T cells were transfected with control (siCTRL) or *p62*-specific (si*p62*) siRNA and Tax-His, together with an NF-κB-luc construct. Luciferase activity was measured and normalized over the corresponding Tax-negative condition. The graph shows results from at least 3 independent experiments. (**f**) Lysates from HEK293T cells transfected with siCTRL or si*p62* and Tax-His were analyzed by western blot. (**g**) Jurkat cells were transfected with increasing amounts of Myc-p62 and an NF-κB-luc construct, followed by transduction with an empty or Flag-Tax-encoding lentivector. Luciferase activity was measured and normalized to the corresponding Tax-negative condition. Values obtained with endogenous p62 were set to 1 and other values are shown as fold change over the “endogenous p62” condition. The graph shows results from 3 independent experiments. (**h**) Lysates from Jurkat cells were analyzed by western blot. ***p < 0.001; **p < 0.01; *p < 0.05 (one-way ANOVA with Bonferroni *post-hoc* test). Full-length blots and gels are presented in Supplementary Fig. S4.

Because p62 has been shown to potentiate NF-κB signaling downstream of TNFα and IL-1 stimulation, we first assessed whether p62 also modulates Tax-induced NF-κB signaling. We first compared Tax-induced NF-κB activation in WT and *p62*^−/−^ MEF cells using a NF-κB-dependent luciferase reporter assay. Interestingly, Tax-dependent NF-κB activation was significantly lower in *p62*^−/−^ MEF cells compared to wild type cells, reaching only 50% of maximal activation (Fig. 2a), thus indicating that p62 is necessary for an optimal activation of NF-κB by Tax. To verify that p62 is specifically involved in Tax-mediated NF-κB activation, and not in other functions of Tax such as the HTLV-1 LTR transactivation, we repeated the experiment using an LTR-dependent reporter. *p62* knock-out did not significantly affect Tax-dependent HTLV-1 LTR activation (Supplementary Fig. S1), thus demonstrating an NF-κB-specific interplay between p62 and Tax. To confirm the involvement of p62 in Tax-mediated NF-κB activation in a luciferase reporter-independent assay, an ELISA assay was performed to quantify the transcriptionally active form of p65 (i.e. able to bind an NF-κB-responsive promoter) in nuclear extracts from Tax-transfected WT and *p62*^−/−^ MEF cells (Fig. 2b and Supplementary Fig. S1 for the controls of cell fractionation). While an increasing level of Tax expression led to an increasing amount of transcriptionally active p65 in WT MEF cells, the increase in transcriptionally active p65 upon Tax expression was undetectable in *p62*^−/−^ MEF cells (Fig. 2b, compare grey bars). As an additional control, we further checked that *p62* knock-out did not affect Tax expression levels (Fig. 2c, compare lanes 2–4 with lanes 6–8).

Then, in order to confirm the importance of p62 in Tax-mediated NF-κB activation, we measured *Il6* expression as an NF-κB target gene in WT or *p62*^−/−^ MEF cells (Fig. 2d). In contrast to WT MEF cells in which Tax expression led to an average 7.5-fold increase in *Il6* mRNA abundance, *Il6* was barely up-regulated upon Tax expression in *p62*^−/−^ MEF cells (Fig. 2d, left and middle panels), confirming that p62 is required for full NF-κB activation by Tax in MEF cells.

Because these results were obtained in murine cells, we then confirmed that p62 was required for an efficient NF-κB activation by Tax in human cells by comparing Tax activity in mock- and *p62*-silenced HEK293T cells (Fig. 2e). In agreement with data from *p62*^−/−^ MEF cells, Tax-dependent NF-κB activation was significantly lower in *p62*-silenced cells compared to mock-silenced cells (Fig. 2e). As a control, we checked that Tax expression levels were also stable upon *p62* silencing in HEK293T cells (Fig. 2f, compare lanes 3 and 6). Results from reporter assays were confirmed with two additional siRNA targeting *p62* (Supplementary Fig. S1), and expression of ectopic *p62* in *p62*-knocked down cells restored full Tax-induced NF-κB activation (Supplementary Fig. S1), further confirming that p62 is responsible for the modulation of Tax function. Of note, the magnitude of p62 effect on Tax-dependent NF-κB activation was similar to the well-documented effect of OPTN, as shown by silencing either *p62* or *OPTN* in HEK293T cells before assaying NF-κB activity (Supplementary Fig. S1). Similar to results obtained in MEF cells, no significant difference was observed for HTLV-1 LTR activation upon *p62* silencing (Supplementary Fig. S1), indicating that p62 is specifically involved in Tax-mediated NF-κB activation in HEK293T cells.

Finally, Tax activity was analyzed in human T cells. Surprisingly, in HTLV-1-infected cells, *p62* silencing did not alter IKK complex activation, as shown by WB analysis of IKKα/β phosphorylation, IκBα phosphorylation and IκBα degradation in C91PL and C8166 cells (Supplementary Fig. S2). Similarly, *p62* silencing in Jurkat T cells did not inhibit Tax-induced NF-κB activation, as shown by WB analysis of IKKα/β phosphorylation and IκBα degradation as well as by a luciferase assay using an NF-κB reporter construct (Supplementary Fig. S2). Importantly, and consistent with a role of p62 in Tax-induced NF-κB activation, expression of ectopic p62 in Jurkat T cells resulted in a 4-fold potentiation of Tax-induced NF-κB activity compared to cells expressing endogenous levels of p62 (Fig. 2g). Of note, the potentiation of Tax-induced NF-κB activation by p62 was confirmed at the level of endogenous IKKα/β phosphorylation in Jurkat T cells, which was specifically detected in Tax-transduced cells and not in mock-transduced cells (Fig. 2h, compare lanes 1-3-5 with lanes 2-4-6), and which increased upon p62 over-expression in the presence of Tax (Fig. 2h, compare lanes 2, 4 and 6). Control western blot as well as flow cytometry analyses confirmed the expression of Tax following transduction as well as the expression of ectopic p62 (Fig. 2h and Supplementary Fig. S2).

Altogether, these results demonstrate that p62 modulates Tax-dependent NF-κB activation in all tested cell types, including human T cells. This effect is specific for NF-κB activation, as p62 is not required for HTLV-1 LTR activation. Mechanistically, this first set of experiments indicates that p62 impacts Tax activity upstream of IKKα/β phosphorylation.

### p62 interacts with the Tax/IKK signalosome in peri-Golgi structures

**Figure 3:**
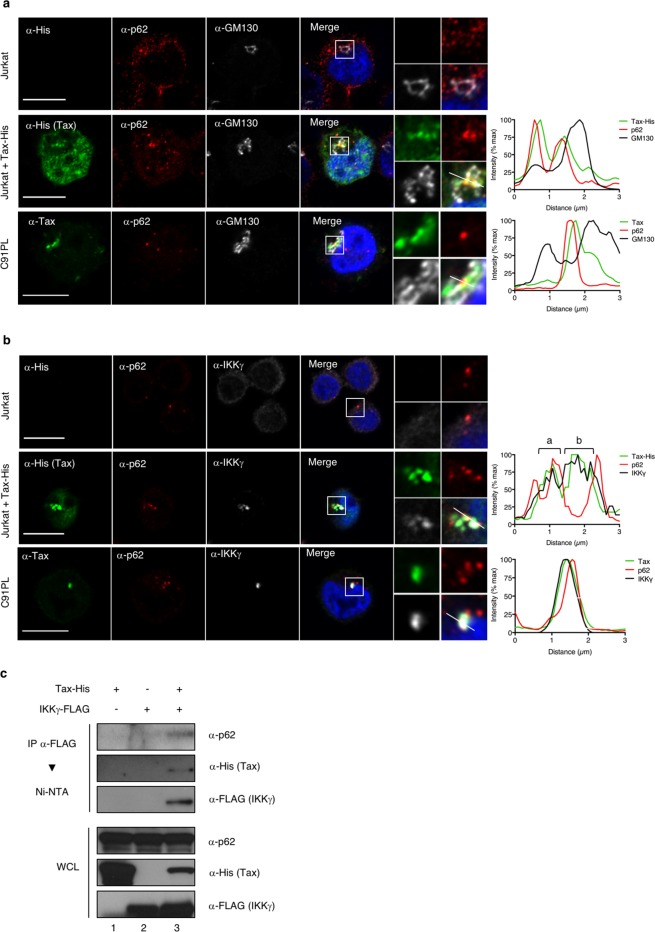
p62 associates with Tax/IKK signalosomes in peri-golgian structures. (**a**,**b**) Jurkat cells transiently expressing Tax-His and HTLV-1 chronically infected C91PL cells were analyzed by confocal microscopy after staining with His- or Tax- (green), p62- (red), and GM130- (**a**, white) and IKKγ-specific (**b**, white) antibodies. Nuclei were counterstained with DAPI (blue). Representative images are shown. Tax, p62, GM130 and IKKγ signals were quantified along the segments represented on the merge panels and plotted on the histogram. Scale bar = 10 μm. (**c**) Lysates from HeLa cells transiently expressing Tax-His and/or IKKγ-FLAG were submitted to a FLAG-immunoprecipitation followed by a His-specific Ni-NTA purification and western blot analyses. Full-length blots are presented in Supplementary Fig. S4.

To explain the potentiation of Tax-dependent IKK activation by p62, and because p62 is known to mediate scaffolding of signaling complexes, we hypothesized that p62 could be associated with the Tax/IKK signalosome, which is known to assemble on golgian membranes. We then analyzed the localization of the Tax/p62 complexes in cells by investigating whether they also stained positive for the GM130 Golgi marker and for IKKγ, used here as a marker of the IKK complex. Confocal microscopy analyses were first performed in Jurkat T cells transiently expressing Tax. Consistent with previous reports, Tax was detected in perinuclear structures in addition to the nucleus (Fig. 3a, second row). Foci of Tax colocalization with endogenous p62 were observed in the cytoplasmic perinuclear region, but not in the nucleus. Perinuclear Tax/p62 speckles were distributed in the vicinity of the Golgi compartment with partial co-localization with GM130 (Fig. 3a, second row, see intensity plot on the right). Similar observations were made in the chronically infected C91PL cell line (Fig. 3a, third row and intensity plot on the right). We next investigated colocalization with IKKγ. As a control, Tax-negative Jurkat T cells exhibited a diffuse distribution of IKKγ and no co-localization between IKKγ and p62 (Fig. 3b, first row). Consistent with previous reports, IKKγ was massively relocalized both in Jurkat cells transiently expressing Tax and in infected C91PL cells, and perfectly colocalized with Tax in perinuclear structures (Fig. 3b, second and third rows). Interestingly, while p62-negative regions of Tax/IKKγ speckles were consistently observed (Fig. 3b, second row, see intensity plot, b), foci of Tax/p62 colocalization were consistently distributed at the periphery of the perinuclear Tax/IKK signalosome, showing an overlap with the IKKγ staining (Fig. 3b, second row, see intensity plot, a; and third row). Taken together, microscopy analyses thus suggest that a fraction of Tax/IKK complexes assembles with p62 in peri-golgian speckles.

To confirm that p62 assembles with Tax/IKK complexes, a biochemical approach was undertaken. HeLa cells were transiently transfected with FLAG-tagged IKKγ and His-tagged Tax constructs and the Tax/IKK signalosome was purified by FLAG-specific immunoprecipitation followed by His-selective Ni-NTA purification (Fig. 3c). FLAG-tagged IKKγ and His-tagged Tax-expressing cells analyzed by this two-step protocol showed that endogenous p62 co-purified with Tax and IKKγ (Fig. 3c, lane 3), demonstrating that p62 is indeed associated with the Tax/IKK signalosome.

### p62 does not allow Tax autophagic degradation at the steady-state

**Figure 4:**
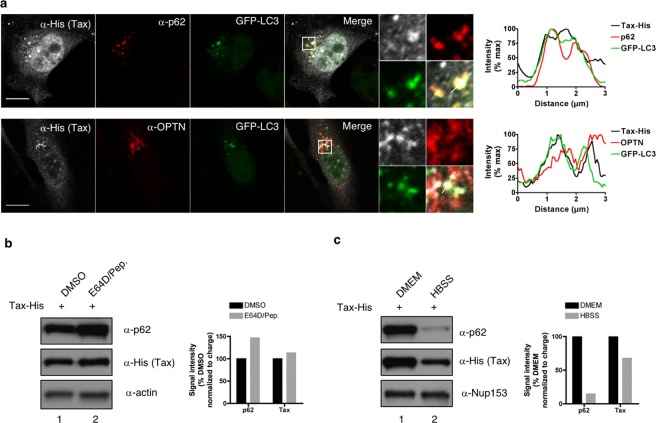
p62 does not allow Tax autophagic degradation at the steady-state. (**a**) HeLa cells stably expressing GFP-LC3 were transfected with Tax-His and analyzed by confocal microscopy after staining with His- (white) and p62- or OPTN-specific (red) antibodies. Representative images are shown. Tax, p62 or OPTN and GFP-LC3 signals were quantified along the segment represented on the merge panel and plotted on the histogram. Scale bar = 10 μm. (**b**,**c**) HeLa cells transiently expressing Tax-His were treated with lysosomal inhibitors (**b**, E64D/Pep.) or starved (**c**, HBSS) before lysis, western blot analyses and quantification. Full-length blots are presented in Supplementary Fig. S4. Blots and graphs show results representative of at least 3 experiments.

Because p62 is known to function as a selective autophagy receptor that allows the recruitment of cargoes to autophagosomal membranes and their degradation following fusion with lysosomes^32,42^, we investigated whether p62 could dually modulate Tax activity by concomitantly recruiting Tax to autophagic degradation. This was tested in HeLa cells stably expressing GFP-LC3, a widely accepted marker of autophagic structures^43^. These cells were transiently transfected with a Tax-encoding plasmid and observed by confocal microscopy (Fig. 4a). Comparison of Tax, p62 and LC3 subcellular distributions showed that foci of Tax/p62 co-localization were GFP-positive (Fig. 4a, see merge panel and intensity plot), indicating that p62 can possibly recruit Tax into LC3-containing structures. Similar observations were made with OPTN (Fig. 4a, see merge panel and intensity plot). To determine whether this possible recruitment of Tax into LC3-containing structures by Tax-interacting selective autophagic receptors might correlate with Tax degradation by lysosomal activity, cells were treated with inhibitors of lysosomal enzymes (E64D and pepstatin) and Tax expression levels were monitored by western blot (Fig. 4b). While lysosomal inhibition stabilized p62 (Fig. 4b, compare lanes 1 and 2 and see quantification), as expected since p62 is a known substrate of autolysosomal degradation, it did not affect Tax expression levels, indicating that at the steady-state, Tax is not degraded in autolysosomes. In addition, starvation-induced autophagy activation (Fig. 4c, “HBSS”) only partially decreased Tax levels compared to p62 levels (Fig. 4c, compare lanes 1 and 2 and see quantification), indicating that starvation-induced autophagy does not efficiently degrade Tax. Taken together, these results suggest that at the steady-state, p62 does not recruit Tax into the autophagic degradation pathway. They thus further support the notion that p62 can potentiate Tax-dependent NF-κB activation.

### p62 directly interacts with Tax via the 170-206 region of p62

**Figure 5:**
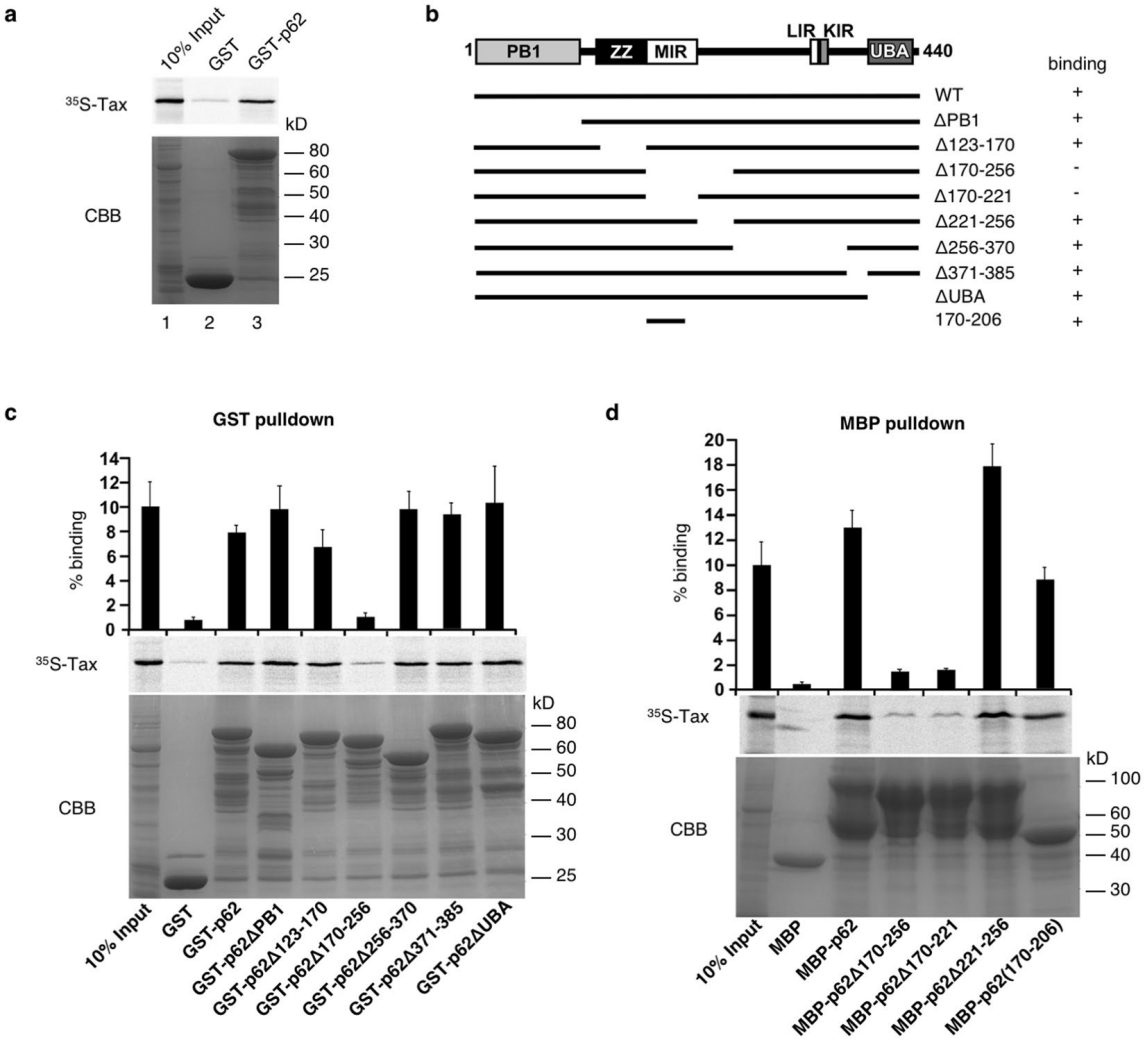
p62 directly interacts with Tax through its 170-206 domain. (**a**) GST and GST-tagged p62 were expressed in bacteria and used for GST pulldown of *in vitro* translated ^35^S-labeled Tax. Inputs as well as eluates were run on SDS-PAGE gels and autoradiography was performed. (**b**) Domain organization of p62 constructs with different deletion used for GST and MBP pulldown assays. The results of the pulldown assays shown in (**c**,**d**) indicate Tax binding ability and are indicated on the right. PB1, Phox and Bem1 domain; ZZ, zinc finger domain; MIR, multiple protein interaction region; LIR, LC3-interacting region; KIR, KEAP1 interacting region; UBA, ubiquitin-associated domain. (**c**,**d**) GST pulldown (**c**) and MBP pulldown assays (**d**) were performed with different p62 constructs. The percentage of input radioactively labeled-Tax bound to p62 was quantified from three independent experiments. CBB, Coomassie brilliant blue-stained SDS-PAGE gel.

We next aimed at gaining insight into the mechanism of p62 potentiation of Tax activity on the NF-κB pathway. To this end, mapping of the interaction domain between p62 and Tax was first undertaken. *In vitro* GST pulldown assays were performed using GST-p62 and *in vitro* translated ^35^S-labeled Tax. This resulted in Tax detection after GST-p62 pulldown (Fig. 5a, lane 3), indicating that Tax and p62 interact directly. Repeating the experiment with GST-tagged deletion mutants of p62 (Fig. 5b), we further showed that the GST-p62Δ170-256 mutant alone was unable to pull down *in vitro* translated Tax, indicating that the 170-256 region of p62 is required for interaction with Tax (Fig. 5c). Using MBP-tagged deletion constructs of p62, we demonstrated that the p62 region 170-221, corresponding to the Multiple protein Interaction Region (MIR), was required for Tax binding (Fig. 5d). In addition, a fragment containing amino acids 170-206 of p62 fused to MBP was sufficient for binding Tax (Fig. 5d). Taken together, these results indicate that Tax interacts directly with p62 via the MIR domain of p62 and more specifically via the 170-206 region.

### p62 binding to ubiquitin is required for p62 potentiation of Tax-mediated NF-κB activation

**Figure 6:**
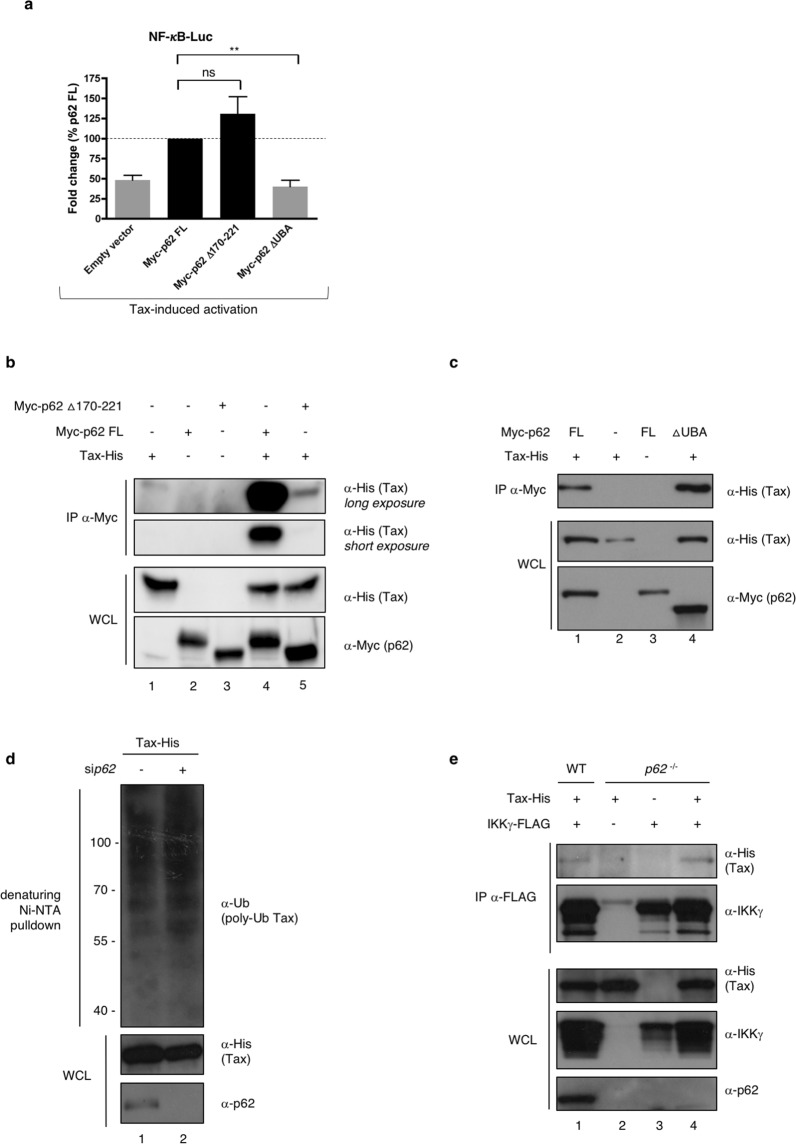
p62 binding to ubiquitin is required for p62 potentiation of Tax-mediated NF-κB activation. (**a**) Jurkat cells were transfected with full-length Myc-p62 (My-p62 FL) or p62 mutants in which the Tax-interacting region (Myc-p62 Δ170-221) or the ubiquitin-binding domain (Myc-p62 ΔUBA) were deleted, and an NF-κB-luc construct, followed by transduction with an empty or Flag-Tax-encoding lentivector. Luciferase activity was measured and normalized to the corresponding Tax-negative condition. Values obtained with full-length ectopic p62 were set to 1 and other values are shown as fold change over the “p62 FL” condition. The graph shows results from 3 independent experiments. (**b**) Lysates from *p62*^−/−^ MEF cells transiently expressing Tax-His and either Myc-tagged full-length p62 (p62FL) or p62 Δ170-221 were immunoprecipitated with a Myc-specific antibody followed by western blot analyses. (**c**) Lysates from *p62*^−/−^ MEF cells transiently expressing Tax-His and either Myc-tagged full-length p62 (p62FL) or p62 ΔUBA were immunoprecipitated with a Myc-specific antibody followed by western blot analyses. (**d**) HeLa cells were transfected with control (−) or *p62*-specific (+) siRNA and Tax-His. Cell lysates were submitted to a His-specific Ni-NTA pulldown in denaturing conditions before western blot analyses. (**e**) Lysates from WT and *p62*^−/−^ MEF cells transiently expressing Tax-His- and FLAG-IKKγ were immunoprecipitated with a FLAG-specific antibody followed by western blot analyses. **p < 0.01; ns, p > 0.05 (one-way ANOVA with Bonferroni *post-hoc* test). Full-length blots are presented in Supplementary Fig. S4.

Following the identification of the domain of p62 required for the interaction with Tax, we hypothesized that a p62 mutant deleted from this domain and thus unable to directly interact with Tax would be unable to potentiate Tax activity on NF-κB. Surprisingly however, when repeating the functional luciferase assay in Jurkat T cells with ectopic p62 Δ170-221, we observed that this p62 mutant did not lose its ability to potentiate Tax-mediated NF-κB activity when compared to full-length (FL) p62 (Fig. 6a), indicating that direct interaction with Tax is dispensable for p62 function on Tax. Because Tax-induced activation of the IKK complex is known to rely on the assembly of a signalosome containing multiple anchored and unanchored ubiquitin chains, and because p62 is known to bind ubiquitin chains via its ubiquitin-associated UBA domain^44,45^, we investigated whether the potentiation of Tax-mediated NF-κB activation by p62 required binding of ubiquitin by p62. We thus included the p62 ΔUBA construct in the luciferase assay. This p62 mutant lost its ability to potentiate Tax-mediated NF-κB activity when compared to the full-length p62 (Fig. 6a), indicating that p62 binding to ubiquitin chains is critical for its ability to enhance Tax-dependent NF-κB signaling. As a control, we further checked that expression levels of both mutants were similar to full-length p62 (Supplementary Fig. S3).

Because assembly of protein complexes *in vitro* may not reflect the processes occurring in cells, we then asked whether the Δ170-221 p62 mutant was able to associate with Tax in cells. Co-immunoprecipitation assays were performed using either the full-length or the Δ170-221 p62 construct. Because p62 is known to polymerize, and in order to avoid rescue of mutant p62 function by polymerization with wild type p62, the experiment was conducted in *p62*^−/−^ MEF cells (Fig. 6b). Interestingly, only a weak association between p62 ∆170-221 and Tax was detected (Fig. 6b, compare lanes 4 and 5), indicating that the 170-221 domain of p62 is indeed necessary for an efficient association with Tax in cells, and thus, that p62 potentiating activity might not correlate with its degree of association with Tax. We then asked whether the UBA domain of p62 could be necessary for its interaction with Tax complexes in cells. Indeed, although it is dispensable for interaction with Tax *in vitro* (see Fig. 5c), it could facilitate recruitment of p62 to ubiquitin-rich Tax complexes in specific cell compartments. Co-immunoprecipitation assays were performed using either the full-length or the ΔUBA p62 construct (Fig. 6c). However, and consistent with Fig. 5c, p62ΔUBA interacted with Tax as efficiently as full-length p62 (Fig. 6c, compare lanes 1 and 4), indicating that p62 binding to ubiquitin is not required for p62 to assemble with Tax complexes.

Tax harbors 10 lysine residues and ubiquitination on lysine 4 to 8 is required for interaction with IKKγ, OPTN and activation of the NF-κB pathway^13,14,16–18,26^. To further confirm that p62 interaction with Tax complexes does not rely on p62 binding to Tax-anchored ubiquitin chains, ubiquitination-defective Tax mutants (Tax-K4-8R, mutated on lysine 4 to 8, and Tax-K1-10R, mutated on all 10 lysine residues) were used. Ubiquitination-defective Tax mutants co-immunoprecipitated with endogenous p62 as efficiently as wild type Tax (Supplementary Fig. S3), indicating that Tax ubiquitination is not required for its interaction with p62, which is consistent with the *in vitro* pulldown experiments. In a control experiment, the antisense protein of HTLV-1 (HBZ) did not immunoprecipitate with p62 (Supplementary Fig. S3), indicating that the Tax/p62 interaction is specific in this assay.

We previously showed that OPTN potentiates Tax activity by stabilizing Tax polyubiquitination through the ubiquitin-binding domain of OPTN^26^. To investigate whether p62 could act in a similar manner, we then compared Tax polyubiquitination in mock- and *p62*-silenced cells (Fig. 6d). Tax polyubiquitination was unaffected by *p62* silencing (Fig. 6d, compare lanes 1 and 2), indicating that in contrast to OPTN^26^, p62 does not modulate Tax polyubiquitination.

Because the p62 ΔUBA protein associates with Tax complexes but is unable to potentiate Tax activity, we further hypothesized that p62 could potentiate Tax activity by allowing the efficient association of the Tax with IKKγ in a ubiquitin-binding-dependent process. Wild type and *p62*^−/−^ MEF cells were transiently transfected with FLAG-tagged IKKγ and His-tagged Tax constructs and cell lysates were submitted to anti-FLAG immunoprecipitation (Fig. 6e). Tax and IKKγ interacted equally efficiently in wild type and *p62*^−/−^ cells (Fig. 6e, compare lanes 1 and 4), indicating that p62 is not required for the association of Tax and IKKγ but is critical for the subsequent activation of the IKK complex.

Taken together, these data support a model in which p62 is recruited to the Tax/IKK complexes in peri-golgian structures, downstream of the association of Tax with IKKγ, and participates in the efficient activation of IKK complexes by a mechanism requiring the binding of ubiquitin chains by p62.

## Discussion

Since the initial identification of IKKγ as a key target of Tax^10–12^, several additional partners required for efficient NF-κB signaling have been characterized by our laboratory and others. These include OPTN and TAX1BP1^26^. Here, using the BioID proteomic approach, we identified Sequestosome-1 (SQSTM-1/p62) as a novel candidate partner of Tax. The recent description of OPTN and TAX1BP1 as members of the SQSTM-1/p62-like selective autophagy receptor (SLR) family and the well-described involvement of p62 in NF-κB regulation prompted us to hypothesize a hijacking of p62 functions for full signaling efficiency by Tax. Our results indicate that in addition to OPTN and TAX1BP1, p62 is indeed also able to potentiate Tax-induced NF-κB signaling. Of note, while ectopic p62 expression in T cells led to a significant potentiation of Tax activity (see Fig. 2g,h), the consequences of p62 knock-out or knock-down differed among the cell lines tested: the decrease in p62 expression did not hamper Tax activity in T cells (see Supplementary Fig. S2), but led to a significantly lower NF-κB activity in MEF or HEK293T cells (see Fig. 2a–f). These observations indicate that while p62 mechanistically modulates Tax functions in all tested cell lines, the requirement of p62 for full Tax-induced NF-κB signaling may be limited to certain cell types and may not apply to T cells. This would be consistent with the emergent view that NF-κB modulation and dynamics may vary in a cell type-specific manner, and in particular may differ between fibroblasts or epithelial cells and immune cells^46^. In T cells, possible additional mechanisms of potentiation (for instance by several members of the SLR family) may hinder the detection of a reduction of Tax activity upon silencing of *p62* alone, in contrast to non-T cells.

Interestingly, Tax interaction with p62 is independent of Tax ubiquitination, and this is a distinctive feature when compared to Tax interaction with OPTN and TAX1BP1. We show that p62 associates with the Tax/IKK signalosome and that binding of p62 to ubiquitin chains is required for p62 to potentiate Tax activity. However, in contrast to OPTN, p62 does not modulate Tax polyubiquitination. p62 is also not required for the efficient assembly of IKKγ with Tax. We therefore propose a model in which p62 participates in the efficient activation of the IKK complex by Tax by a mechanism requiring the scaffolding of ubiquitin chains, in agreement with the known functions of p62 in NF-κB signaling.

Indeed, p62 is a multi-functional scaffold protein regulating NF-κB activation downstream of TNFR, IL1R, CD40 and TCR^36^. Its scaffold properties mainly arise from its ability to bind ubiquitin chains through its C-terminal UBA domain^47^. p62 shows high affinity for linear M1-linked polyubiquitin chains, and a weaker but significant affinity for K63-linked chains, while the affinity for K48 chains is low^48^. The link between ubiquitin binding by p62 and NF-κB signaling was first identified following the analysis of p62 mutations present in Paget’s disease of bone (PDB). This disorder results from defects in osteoclast differentiation and/or activity, which are controlled by the NF-κB pathway. p62 mutations in PDB patients are commonly affecting the UBA domain, indicating that ubiquitin binding by p62 could modulate NF-κB signaling^49^. The UBA domain of p62 was further shown to modulate TRAF6 polyubiquitination by influencing TRAF6 E3 ligase activity^34^.

The domain of p62 that engages Tax by direct interaction (amino acids 170 to 221) encompasses the MIR (for Multiple Protein Interaction Region) through which several proteins are known to bind p62, including the LIM domain protein Ajuba^50^, p38 MAPK^51^, a familial amyotrophic lateral sclerosis (ALS) mutant of superoxide dismutase (SOD1)^52^ and the autophagy adapter ALFY^53^. Hence, this region functions as an important protein-protein interaction domain. This direct interaction, which does not require Tax ubiquitination, contrasts with Tax/IKKγ and Tax/OPTN interactions for which Tax ubiquitination is essential^26^. Interestingly, p62 has been described to interact with other viral proteins independently of their ubiquitination, such as with the capsid protein of Sindbis virus^54^ and with the Tat transactivator of human immunodeficiency virus type 1^55^, leading to the autophagic clearance of these viral proteins. However, and in contrast to our work, these interactions were analyzed in cells and not *in vitro*, so that indirect interactions cannot be excluded in those settings. Hence, Tax is the first direct, ubiquitin-independent viral partner of p62 to be reported.

**Figure 7:**
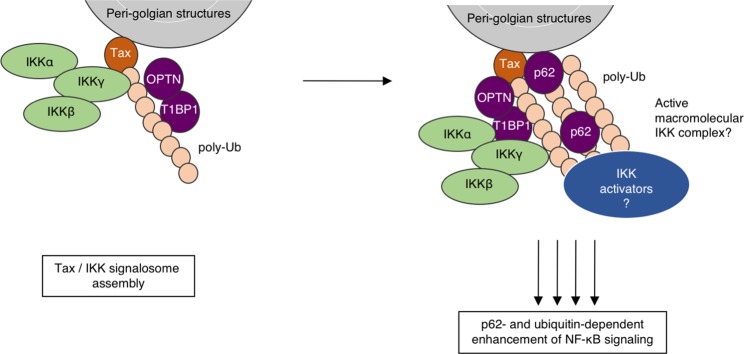
Involvement of ubiquitin binding by p62 in Tax-induced NF-κB activation: working model. p62 associates with Tax in peri-golgian structures, downstream of the formation of Tax/IKK complexes, and participates in the efficient activation of the IKK complex by a mechanism requiring binding to ubiquitin chains. See text for further details. T1BP1: TAX1BP1. Artwork is a derivative of Servier Medical Art (https://smart.servier.com/), used under CC BY 3.0 FR (https://creativecommons.org/licenses/by/3.0/), and includes modifications in shapes, colors and disposition of the original material.

To our surprise however, a p62 mutant in which the Tax-interaction region is deleted, and which thus can no longer directly interact with Tax (see Figs 5 and 6b), is still able to efficiently potentiate Tax activity (see Fig. 6a). Our data thus support a two-step mechanism (Fig. 7): (i) association of Tax with IKKγ in a p62-independent process (see Fig. 6e) that initiates the assembly of ubiquitin-rich IKK complexes and (ii) recruitment of p62 by both direct interaction with Tax and indirect binding to ubiquitin chains, the latter being necessary to enhance Tax signaling. Because Tax induces the synthesis of anchored and unanchored polyubiquitin chains, our data indicate that p62 could interact with polyubiquitin chains produced upon Tax expression and retain them in close proximity to the IKK signalosome for full activation. p62-mediated scaffolding of Tax-induced ubiquitin chains combined with p62 ability to oligomerize would possibly facilitate the organization of active macromolecular IKK complexes favorable to IKK *trans-*autophosphorylation, and/or the recruitment of the activating kinases of IKK such as MEKK3, which interacts with p62 through p62 PB1 domain^38^, although the requirement of MEKK3 for Tax-induced NF-κB activation is still under debate^22^.

In addition, although TRAF6 was initially described as dispensable for Tax-induced NF-κB activation^56^, Tax has also been demonstrated to interact with TRAF6 and to promote TRAF6 self-ubiquitination in an IKKγ-dependent manner, a process that was proposed to play a role in Tax-induced NF-κB activation^57,58^. Whether Tax exploits p62 ability to increase TRAF6 self-ubiquitination^34^ for efficient NF-κB signaling remains to be investigated. In IL-1-induced NF-κB signaling, p62 has recently been shown to compete with the de-ubiquitinase YOD1 for the binding to TRAF6^59^. Thus, association of p62 with the Tax/IKK signalosome could similarly help it to escape negative regulatory mechanisms such as editing of polyubiquitin chains by the A20/Itch complex^27^ or de-ubiquitination by CYLD or USP20^58,60,61^, although this needs to be tested.

Our results indicate that while p65 activity induced by Tax is totally abrogated in *p62*-deficient cells, a residual activity of the synthetic NF-κB reporter construct (which responds to several members of the NF-κB family in addition to p65) is detected in these cells. This suggests that p62 may be specifically involved in the activation of the canonical (*i.e* p65-dependent) NF-κB pathway by Tax, and may not be required for activation of the non-canonical (*i.e* p65-independent) pathway.

Recent work by Paul *et al*. showed that p62 scaffolds the MALT1-BCL10-TRAF6-IKK signalosome in so-called cytosolic POLKADOTS (punctate and oligomeric killing or activating domains transducing signals) downstream of TCR activation in T cells^40^. These POLKADOTS are associated with autophagic structures involved in the degradation of BCL10 and termination of signaling^62^. In our study, assessment of Tax/p62 localization in GFP-LC3-expressing cells showed that these complexes may be localized at LC3-positive structures. However, Tax expression levels were unaffected by lysosomal inhibition, indicating that at the steady-state, Tax is not degraded by an autophagic pathway. This is in agreement with the fact that Tax has been shown to inhibit autophagic degradation in infected cells through inhibition of the fusion between autophagosomes and lysosomes^63^. Thus, despite its functions as a selective autophagy receptor, p62 could indeed be a positive modulator of Tax-induced NF-κB activation.

Taken together, our results identify p62 as a new modulator of Tax activity on NF-κB and support a ubiquitin-dependent scaffolding role for p62 in this process, further highlighting the importance of ubiquitin in the signaling activity of the viral Tax oncoprotein. Together with the previously published observations that Tax exploits OPTN and TAX1BP1 functions for NF-κB signaling, these results indicate that Tax might hijack the functions of the Sequestosome-1/p62-like selective autophagy receptor family for its signaling activity.

## Methods

### Cell culture

HeLa and HEK293T cells were obtained from the American Type Culture Collection (ATCC). U2OS cells were a kind gift from Dr. Michel Bornens. Wild-type and *p62* knockout (*p62*^−/−^) MEF cells were kindly provided by Dr. Toru Yanagawa. These cells were grown in high glucose, GlutaMAX Dulbecco’s modified Eagle medium (DMEM, Gibco) supplemented with 10% fetal bovine serum (Biosera) and antibiotics (100 U/ml penicillin and 100 μg/ml streptomycin, Gibco). Non-infected Jurkat T cells (ATCC) and HTLV-1 chronically infected C8166, HuT102 and C91PL T cells (kind gifts from Dr. Antoine Gessain) were grown in GlutaMAX Roswell Park Memorial Institute medium (RPMI, Gibco) supplemented with 10% fetal bovine serum (Biosera) and antibiotics (100 U/ml penicillin and 100 µg/ml streptomycin, Gibco). All cells were maintained at 37 °C in 5% CO_2_. In order to induce autophagy, cells were cultivated in Hank’s Balanced Salt Solution (HBSS) for 6 hours.

### Constructs and siRNA

pSG5M vectors encoding His-tagged Tax, Tax-K4-8R, Tax-K1-10R have been previously described^13^. The Tax-His coding sequence was amplified by PCR and tagged with BirA* by cloning between *Eco*RI and *Hind*III sites into the Myc-BioID2-MCS vector (Addgene #74223, from Dr Kyle Roux^41^). The HBZ-SP1 coding sequence was amplified from the pcDNA3.1-Myc-His vector (a kind gift from Dr. Jean-Michel Mesnard)^64^ and cloned into the pSG5M backbone with a C-terminal His-tag. Flag-Tax-encoding lentivectors have been described previously^65^. Myc-p62 constructs cloned into the pCMV-Myc-Gat backbone were kindly provided by Dr. Anne-Sophie Nicot and Dr. Laurent Schaeffer. Myc-p62FL and Myc-p62ΔUBA constructs cloned into pcDNA3-Myc backbone were kindly provided by Dr. Jorge Moscat^34^. The Myc-p62 Δ170-221 was generated from Myc-p62FL by site-directed mutagenesis (QuikChange II XL Site-Directed Mutagenesis Kit, Agilent Technologies, #200521) using the following primers: forward primer CCAAGCTCGCATTCCCCAGCCCACGTCCTCC; reverse primer GGAGGACGTGGGCTGGGGAATGCGAGCTTGG. The p62 expression vectors used for GST pulldown, or their ENTR constructs (pDEST15-p62, pDEST15-p62ΔPB1, pDEST15-p62Δ123-170, pDEST15-p62Δ170-256, pDEST15-p62Δ256-370, pDEST15-p62Δ371-385 and pDEST15-p62ΔUBA), or used for MBP pulldown (pTH1-p62, pTH1-p62Δ170-256, pTH1-p62Δ170-221, pTH1-p62Δ221-256 and pTH1-p62(170-206)) have been described before^53^. The NF-κB-luciferase and HTLV-1-LTR-luciferase reporter gene plasmids have been previously described^66^. The FLAG-tagged IKKγ expression vector was used previously^66^.

Non-targeting siRNA and *p62*-specific siRNA were purchased from Dharmacon (ON-TARGET plus Human SQSTM1) and from Ambion (Silencer Select Human SQSTM1, siRNA ID #s16961 and #s16962, referred to here as si*p62* #2 and si*p62* #3). *OPTN*-specific siRNA were from Sigma-Aldrich.

### Antibodies and reagents

The following antibodies were used: anti-His (ab9136 or ab18184, Abcam or sc-804, Santa Cruz), anti-Tax1 (Tab172, NIH), anti-p62 (GP62-C, Progen), anti-OPTN (100000, Cayman), anti-GM130 (610823, BD), anti-IKKγ (611306, BD), anti-phosphorylated IKKα/β (2078, Cell Signaling Technology), anti-IκBα (4814, Cell Signaling Technology), anti-phosphorylated IκBα (9246, Cell Signaling Technology), anti-FLAG (M2, Sigma-Aldrich), anti-Myc (4A6, Millipore), anti-Ub (P4D1, sc-8017, Santa Cruz), anti-actin (AC-74, Sigma-Aldrich), anti-Nup153 (147050002, Covance). HRP-Streptavidin was from Sigma (RAB-HRP3). For control immunoprecipitation, normal guinea pig IgG (sc-2711, Santa-Cruz) were used. For inhibition of lysosomal degradation, E64D and pepstatin (Sigma) were both used at 10 µg/mL.

### Transient transfections and transduction

HEK293T and HeLa cells were transfected using either Polyfect or Effectene (Qiagen), or with DreamFect Gold (OZ Biosciences). MEF and U2OS cells were transfected using Lipofectamine 2000 (Life Technologies). For siRNA transfections, the Lipofectamine RNAiMax reagent (Life Technologies) was used, while Lipofectamine 2000 (Life Technologies) was used for siRNA and DNA co-transfection. Jurkat cells, C91PL cells and C8166 cells were transfected using the Neon electroporation system (Thermo Fisher). When indicated, Jurkat cells were transduced using Flag-Tax-expressing lentiviruses, as described previously^65^.

### BioID procedure and mass spectrometry analyses

HEK293T cells were transfected with BirA*-encoding vectors for 24 h and treated with biotin (50 µM, Sigma) for 18 h. BioID streptavidin pulldown was performed on whole cell lysates as described previously^67^ using Dynabead MyOne Streptavidin C1 (ThermoFisher Scientific). After the washing steps, beads were washed twice in 50 mM NH_4_HCO_3_. Bound proteins were reduced with 200 mM DTT and alkylated with 200 mM Iodoacetamide. Digestion was performed with trypsin enzyme (Promega) at a ratio 1/100 overnight at 37 °C. After desalting on C18 cartridges (Harvard Apparatus), samples were analyzed qualitatively or in a Label Free quantitation strategy using an Ultimate 3000 nano-RSLC (Thermo Scientific, San Jose California) coupled on line with a Q Exactive HF mass spectrometer via a nano-electrospray ionization source (Thermo Scientific, San Jose California). Samples were injected and loaded on a C18 Acclaim PepMap100 trap-column 75 µm ID x 2 cm, 3 µm, 100 Å, (Thermo Scientific) for 3.0 minutes at 5 µL/min with 2% ACN, 0.05% TFA in H_2_O and then separated on a C18 Acclaim Pepmap100 nano-column, 50 cm × 75 µm i.d, 2 µm, 100 Å (Thermo Scientific) with a 60-minute linear gradient from 3.2% to 40% buffer B (A: 0.1% FA in H_2_O, B: 100% ACN, 0.1% FA) and then from 40 to 90% of B in 2 min, hold for 10 min and returned to the initial conditions in 1 min for 14 min. The total duration was set to 90 minutes at a flow rate of 300 nL/min. The oven temperature was kept constant at 40 °C.

Samples were analysed in triplicate with TOP20 HCD method: MS data were acquired in a data-dependent strategy selecting the fragmentation events based on the 20 most abundant precursor ions in the survey scan (350-1600 Th). The resolution of the survey scan was 60,000 at m/z 200 Th and for MS/MS scan the resolution was set to 15,000 at m/z 200 Th. The Ion Target Value for the survey scans in the Orbitrap and the MS/MS scan were set to 3E6 and 1E5 respectively and the maximum injection time was set to 60 ms for MS and MS/MS scan. Parameters for acquiring HCD MS/MS spectra were as follows: collision energy = 27 and an isolation width of 2 m/z. The precursors with unknown charge state, charge state of 1 and 8 or greater than 8 were excluded. Peptides selected for MS/MS acquisition were then placed on an exclusion list for 30 s using the dynamic exclusion mode to limit duplicate spectra.

Data files were then analyzed with Proteome Discover 2.1 or with with Proteome Discover 2.2 using the SEQUEST HT algorithm against the Swissprot Human database (2018-09 release, 20332 sequences), complemented with the Uniprot HTLV sequences (2018-09 release, 3298 sequences) and the BirA and streptavidin sequences. Precursor mass tolerance was set at 10 ppm and fragment mass tolerance was set at 0.02 Da, and up to 2 missed cleavages were allowed. Oxidation (M), acetylation (Protein N-terminus) were set as variable modification, and Carbamidomethylation (C) as fixed modification. Peptides and proteins were filtered with a false discovery rate (FDR) at 1% using percolator. For qualitative analysis, proteins specifically identified in the BirA*-Tax-transfected cells and not in the BirA*-transfected control cells were considered. For quantitative analysis, protein quantification was done by the Label Free Quantification (LFQ) approach, and LFQ abundance values were obtained for each sample, normalized to the total peptide amount. The abundance ratio [BirA*-Tax/BirA*] was calculated as well as the adjusted p-value. The numbers presented in Fig. 1e include the SEQUEST HT score. The highest this score is, the better the protein is identified.

### Imunofluorescence microscopy

Cells were fixed in 10% formalin solution (HT5011, Sigma-Aldrich) or ice-cold methanol (for Fig. 1b), washed and permeabilized in PBS containing 0.5% Triton X-100. After saturation in PBS containing 0.2% Tween (PBS-T) and 5% milk, cells were incubated with primary antibodies for 1 h at room temperature. After three washes in PBS-T, cells were incubated with Dylight 488 anti-mouse IgG (DI-2488, Vector), Dylight 488 anti-rabbit IgG (ab98488, Abcam), Dylight 549 anti-mouse IgG (DI-2549, Vector), AlexaFluor 647 anti-mouse (715-605-150, Jackson), Alexa Fluor 555 anti-guinea pig IgG (ab150186, Abcam) or AlexaFluor 647 Streptavidin (S21374, ThermoFisher Scientific) for 1 h at room temperature. After three washes in PBS-T, coverslips were then mounted on glass slides in Fluoromount-G medium containing DAPI (0100-20, Southern Biotech).

GFP-LC3 HeLa cells were plated in 6-well plates and transfected using DreamFect Gold (OZBiosciences). Twenty-four hours after transfection, cells were transferred onto glass coverslips. The next day, cells were fixed in 2% paraformaldehyde, washed and permeabilized using Triton X100. After saturation in PBS supplemented with 1% BSA, coverslips were incubated overnight at 4 °C with primary antibodies diluted in saturation buffer. After washing, coverslips were incubated for 45 min at 4 °C with secondary antibodies diluted in saturation buffer. Coverslips were then mounted onto glass slides using Dako Fluorescence mounting medium.

Samples were examined under a Leica spectral SP5 confocal microscope equipped with a 63x 1.4–0.6 oil-immersion objective using the LAS-AF software, or under a Zeiss LSM800 confocal microscope equipped with a 63x 1.4 oil immersion objective using the ZEN software. For Fig. 1b, samples were observed under an AxioImager.Z1 microscope (Zeiss) equipped with a 63x/1.4 Plan Apochromat oil-immersion objective using the Metamorph software. Images were processed with ImageJ^68^.

### Reporter gene assays

HEK293T and Jurkat cells were transfected with the NF-κB-luciferase or HTLV-1-LTR-luciferase reporter vectors together with the indicated plasmids or siRNA. A *Renilla* luciferase reporter vector (phRG-TK, Promega) was used as an internal control to normalize for transfection efficiency. Cells were harvested 24 h after transfection and assayed for luciferase activity using the Dual-Luciferase Reporter Assay System (Promega), according to the manufacturer’s instructions, and a Mithras multimode plaque reader luminometer (Berthold).

### NF-κB (p65) transcription factor ELISA assay

After transfection, cells were fractionated using the following buffers: buffer A for cytoplasm extraction (0.2% Igepal-630, 10 mM HEPES, 10 mM KCl, 1.5 mM MgCl_2_, 0.5 mM DTT, 100 mM NaF, 2 mM Na_3_VO_4_, supplemented with protease inhibitors [Complete, Roche]) and buffer B for nuclear extraction (50 mM Tris-HCl pH 8.0, 400 mM NaCl, 5 mM EDTA, 1% Igepal-630, 0.2% SDS, 1 mM DTT, 100 mM NaF, 2 mM Na_3_VO_4_, supplemented with protease inhibitors). Protein quantification was performed using the detergent-compatible Bradford assay kit (Pierce, #23246). Active p65 was quantified from 2 µg of WT and p62^−/−^ MEF cell nuclear extracts using the Cayman Chemical assay kit (#10007889), following the supplier’s instructions.

### Western blot analyses, immunoprecipitation, Ni-NTA purification and GST and MBP pulldown assays

For western blots, cells were lysed for 20 min on ice in the following buffer: 50 mM Tris pH 7.4, 150 mM NaCl, 1% NP40, 0.25% sodium deoxycholate, complemented with protease inhibitors (Complete, Roche) and 1 mM phenylmethylsulfonyl fluoride (PMSF). After centrifugation and protein concentration determination (Bradford, Biorad), proteins were analyzed by SDS-PAGE. Alternatively, for Fig. 2h, cells were lysed in Laemmli buffer before SDS-PAGE analysis.

For immunoprecipitation, cells were lysed for 20 min on ice in the following buffer: 50 mM Tris pH 7.4, 150 mM NaCl, 1% NP40, complemented with protease inhibitors (Complete, Roche) as well as the phosphatase inhibitors NaF (100 mM) and Na_3_VO_4_ (2 mM), and then scraped from Petri dishes. Cell lysates were passed through 26G^1/2^ needles, centrifuged, and protein concentration was quantified using the Bradford method (Biorad). Equal quantities of proteins were immunoprecipitated overnight at 4 °C with anti-p62, anti-Myc antibodies or normal guinea pig IgG and *Staphylococcus aureus* Protein G sepharose beads (GE Healthcare). After three washes in lysis buffer, proteins were eluted and analyzed by SDS-PAGE.

For anti-FLAG immunoprecipitation followed by His purification, cell lysates were incubated with an anti-FLAG M2 affinity gel (A2220, Sigma-Aldrich). After three washes in lysis buffer, bound proteins were eluted with 3X FLAG peptide (F4799, Sigma-Aldrich). His-specific purification was then performed using His-select HF agarose beads (H0537, Sigma-Aldrich). After three washes in lysis buffer, proteins were eluted and analyzed by SDS-PAGE.

For ubiquitination assay, denaturing Ni-NTA pulldown was performed as described previously^15^.

For GST and MBP pulldown assays, GST and GST-tagged proteins were expressed in *E. coli* soluBL21 (Amsbio) and immobilized on glutathione-coupled Sepharose beads (Glutathione-Sepharose 4 Fast Flow; GE Healthcare). MBP and MBP-tagged proteins were expressed in *E. coli* BL21(DE3) (Amsbio) and immobilized on amylose resin (New England Biolabs). Pulldown assays with *in vitro* translated [^35^S]-labeled proteins (here HTLV-1 Tax) were performed as described previously^42^.

For SDS-PAGE analysis, proteins were separated on 10% or 12% Bis-Tris gels or on 10% or 12% TGX stain-free gels (Criterion, Biorad) and transferred onto PVDF membranes (Immobilon P, Millipore). After incubation in TBS-Tween (Sigma-Aldrich) containing 5% milk, membranes were incubated in primary antibodies. Horseradish peroxidase-conjugated anti-mouse IgG (NA9310, GE Healthcare or #32430, Thermo Fisher), anti-rabbit IgG (NA9340, GE Healthcare or #32460, Thermo Fisher), and anti-guinea pig Ig (P0141, Dako) were used as secondary antibodies, before revelation with ECL Prime (Amersham), ECL Select (Amersham) or SuperSignal West Femto (Thermo Fisher) substrates. Signals were quantified using the ImageJ software^68^.

### RT-PCR assay

Total RNAs were obtained by Trizol extraction. After treatment with DNAse (DNA-free™ DNA Removal Kit, Invitrogen), 500 ng of total RNA were converted to cDNA using the RevertAid First Strand cDNA Synthesis Kit (Thermo Scientific), following the supplier’s instructions with OligodT primers. A volume of 2 μl of cDNA was then used in a PCR reaction (25 µl reaction volume) containing 1X PCR buffer (Invitrogen), 0.4 μM of forward and reverse primers, 0.2 mM dNTP mix, 1.5 mM MgCl_2_, and 0.5 U of *Taq* DNA polymerase (Invitrogen). The *gapdh* primers from the RevertAid First Strand cDNA Synthesis Kit were used to amplify *gapdh* cDNAs (496 bp). The following primer pair was used to amplify *Il6* cDNAs (460 bp): forward 5’-GACTTCACAGAGGATACCACTC and reverse 5’-GTCCTTAGCCACTCCTTCTG. PCR products were visualized after agarose gel electrophoresis. Signals were quantified using the Image Lab software (Biorad).

### Flow cytometry

To estimate the transduction efficiency with the GFP-encoding lentivector, cells were fixed in PBS containing 4% paraformaldehyde and analyzed on a MACSQuant cytometer (Miltenyi). Analyses were performed under the FlowJo software.

### Statistical analyses

Data are presented as mean ± SEM. One-way ANOVA with Bonferroni *post-hoc* test was used to compare means (GraphPad Prism). Differences between means were considered significant when the p-value was less than 0.05. ***p < 0.001; **p < 0.01; *p < 0.05; ns, p > 0.05.

## Supplementary information

Supplementary figures

## Data Availability

The datasets generated during the current study are available from the corresponding author on reasonable request.
