# Supplementary material for: SQSTM-1/p62 potentiates HTLV-1 Tax-mediated NF-κB activation through its ubiquitin binding function
Source: Sci Rep. 2019 Nov 5;9:16014. doi: 10.1038/s41598-019-52408-x (PMC6831704; doi:10.1038/s41598-019-52408-x)
Supplement: Supplementary file 1 — Supplementary figures [file 41598_2019_52408_MOESM1_ESM.pdf]

SQSTM-1/p62 potentiates HTLV-1 Tax-mediated NF-κB activation through its ubiquitin binding function

Aurélien Schwob, Elodie Teruel, Louise Dubuisson, Florence Lormières, Pauline Verlhac, Yakubu Princely Abudu, Janelle Gauthier, Marie Naoumenko, Fanny-Meï Cloarec-Ung, Mathias Faure, Terje Johansen, Hélène Dutartre, Renaud Mahieux, Chloé Journo

Supplementary figures

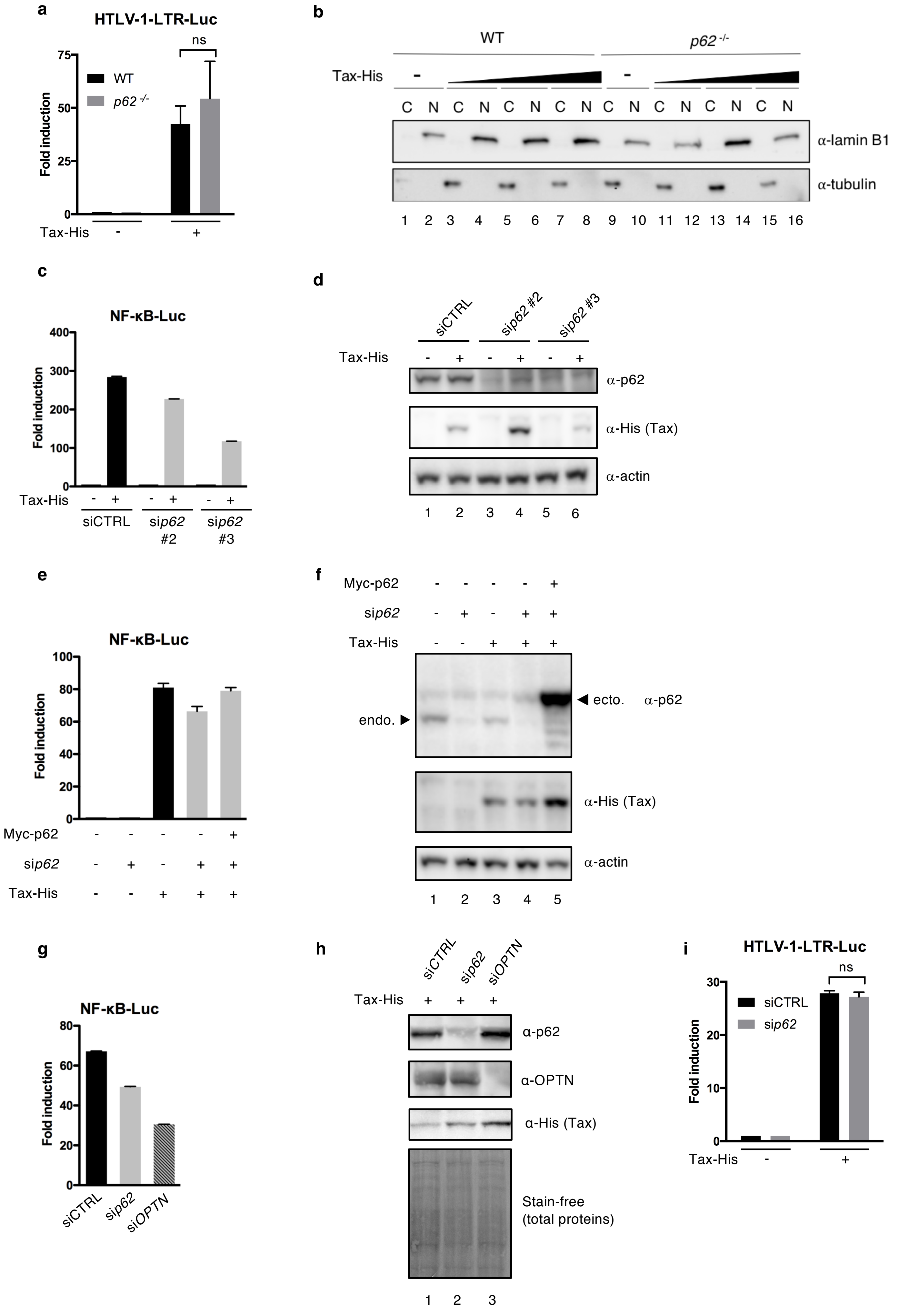

Supplementary Figure S1. p62 potentiates Tax-dependent NF-κB activation (relative to Fig. 2).
See legend on next page.

Supplementary Figure S1. p62 potentiates Tax-dependent NF-κB activation (relative to Fig. 2).
(**a**) Wild type (WT) and *p62*$^{-/-}$ MEF cells were transfected with Tax-His, together with an HTLV-1-LTR-luc construct. Luciferase activity was measured and normalized over the corresponding Tax-negative condition. The graph shows results from at least 3 independent experiments. (**b**) Lysates from WT and *p62*$^{-/-}$ MEF cells were analyzed by western blot after cell fractionation. C: cytoplasmic fractions. N: nuclear fractions. (**c**) HEK293T cells were transfected with control (siCTRL) or two additional *p62*-specific (si*p62* #2 and si*p62* #3) siRNA and Tax-His, together with an NF-κB-luc construct. Luciferase activity was measured and normalized over the corresponding Tax-negative condition. (**d**) Lysates from HEK293T cells transfected with siCTRL, si*p62* #2, si*p62* #3 and Tax-His were analyzed by western blot. **(e)** HEK293T cells were transfected with control (-) or *p62*-specific (+) siRNA, Tax-His and ectopic Myc-p62, as indicated, together with an NF-κB-luc construct. Luciferase activity was measured and normalized over the corresponding Tax-negative condition. (**f**) Lysates from HEK293T cells transfected with siCTRL, si*p62*, Tax-His and ectopic Myc-p62 were analyzed by western blot. (**g**) HEK293T cells were transfected with control (siCTRL), *p62*-specific (si*p62*) or *OPTN*-specific (si*OPTN*) siRNA and Tax-His, together with an NF-κB-luc construct. Luciferase activity was measured and normalized over the corresponding Tax-negative condition. The graph shows the result from a representative experiment repeated twice. (**h**) Lysates from HEK293T cells transfected with siCTRL, si*p62* or si*OPTN* and Tax-His were analyzed by western blot. (**i**) HEK293T cells were transfected with control (siCTRL) or *p62*-specific (si*p62*) siRNA and Tax-His, together with an HTLV-1-LTR-luc construct. Luciferase activity was measured and normalized over the corresponding Tax-negative condition. The graph shows results from at least 3 independent experiments. ns, $p>0.05$ (one-way ANOVA with Bonferroni *post-hoc* test). Full-length blots are presented in Supplementary Figure S4.

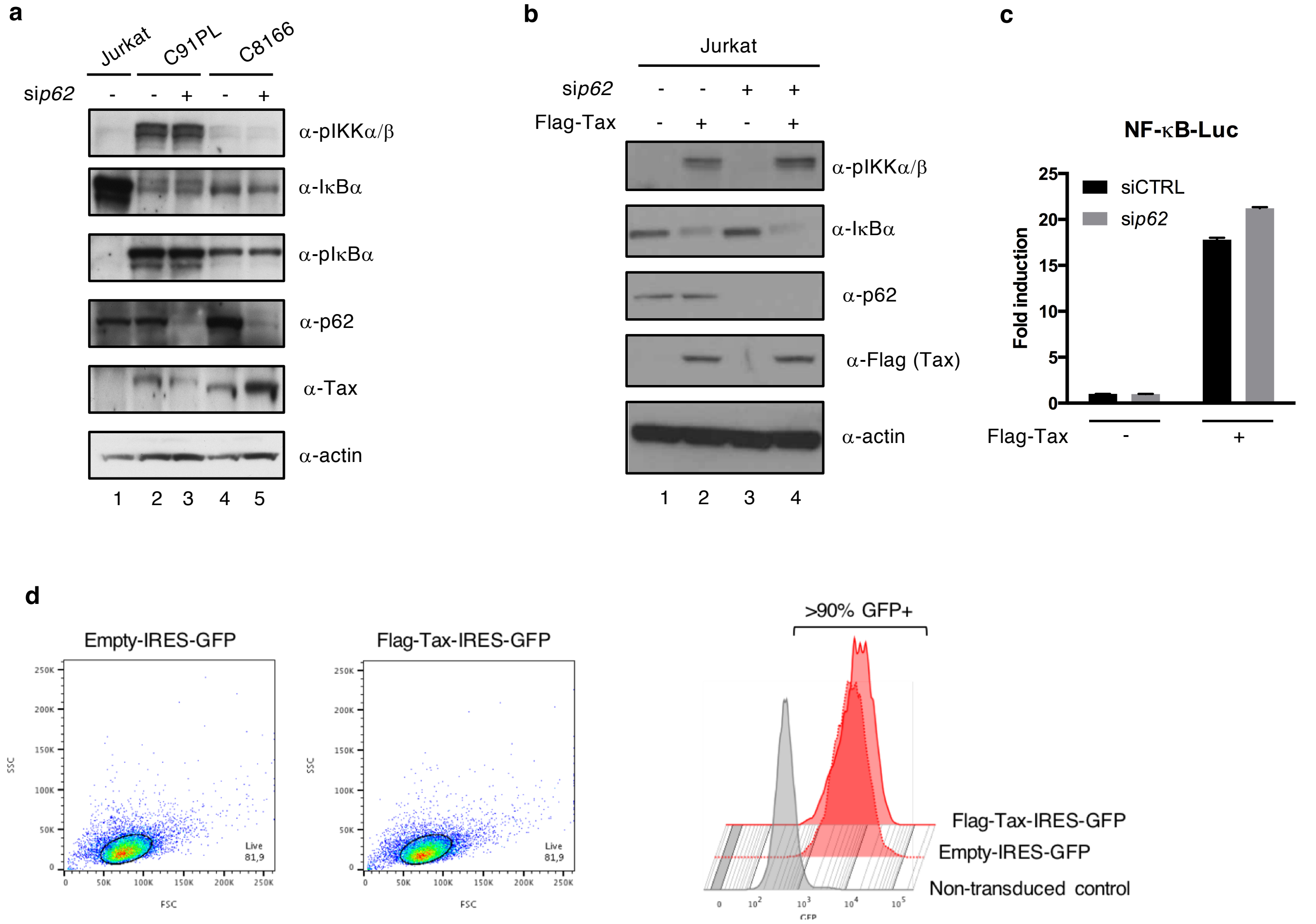

Supplementary Figure S2. Analysis of p62 activity on Tax-dependent NF-κB activation in T cells (relative to Fig. 2).

(**a**) Non-infected T cells (Jurkat) and HTLV-1 chronically infected cells (C91PL and C8166) were transfected with control (-) or *p62*-specific (+) siRNA for 48 hours. Cell lysates were analyzed by western blot. (**b-c**) Jurkat T cells were transfected with control (-) or *p62*-specific (+) siRNA and an NF-κB-luc construct, followed by transduction with an empty or Flag-Tax-encoding lentivector. (**b**) Lysates were analyzed by western blot. (**c**) Luciferase activity was measured and normalized to the corresponding Tax-negative condition (set to 1). The graph is representative from 2 independent experiments. (**d**) Jurkat cells were transfected with increasing amounts of Myc-p62 and an NF-κB-luc construct, followed by transduction with an empty or Flag-Tax-encoding lentivector, which also encodes GFP after an IRES. Cells were analyzed by flow cytometry to determine the proportion of live cells (FCS/SSC panel) and of GFP-positive (GFP+) transduced cells. Full-length blots are presented in Supplementary Figure S4.

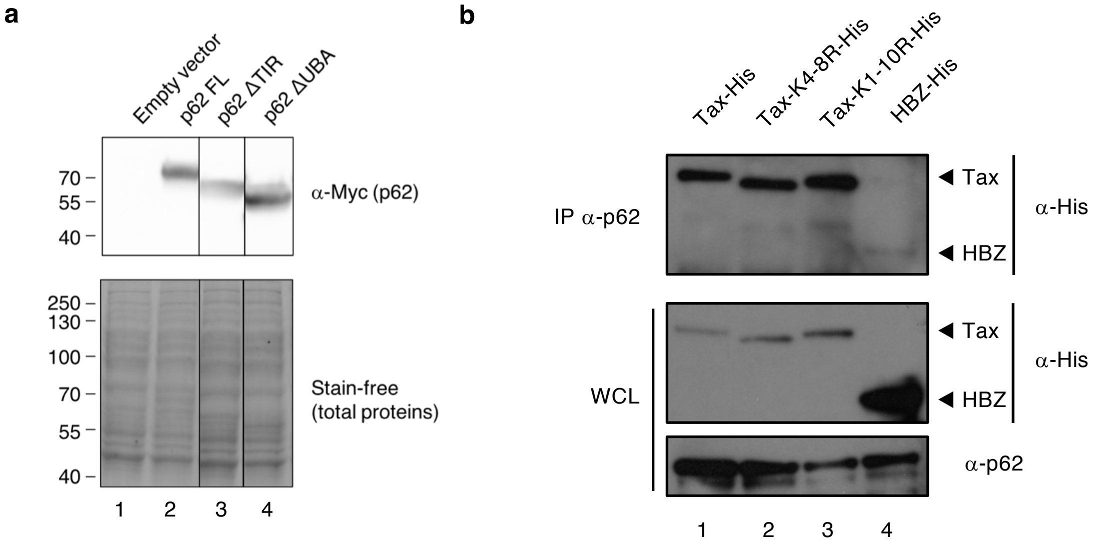

Supplementary Figure S3. p62 binding to ubiquitin is required for p62 potentiation of Tax-mediated NF-κB activation (relative to Fig. 6).

(**a**) Cells were transfected with full-length Myc-p62 (My-p62 FL) or p62 mutants in which the Tax-interacting region (Myc-p62 Δ170-221) or the ubiquitin-binging domain (Myc-p62 ΔUBA) were deleted, and cell lysates were analyzed by western blot. The vertical lines separate several parts of a single membrane. (**b**) Lysates from HeLa cells transiently expressing wild type Tax-His, ubiquitination-defective Tax-K4-8R-His and Tax-K1-10R-His or HBZ-His were immunoprecipitated with a p62-specific antibody followed by western blot analyses. Full-length blots are presented in Supplementary Figure S4.

Figure 1a

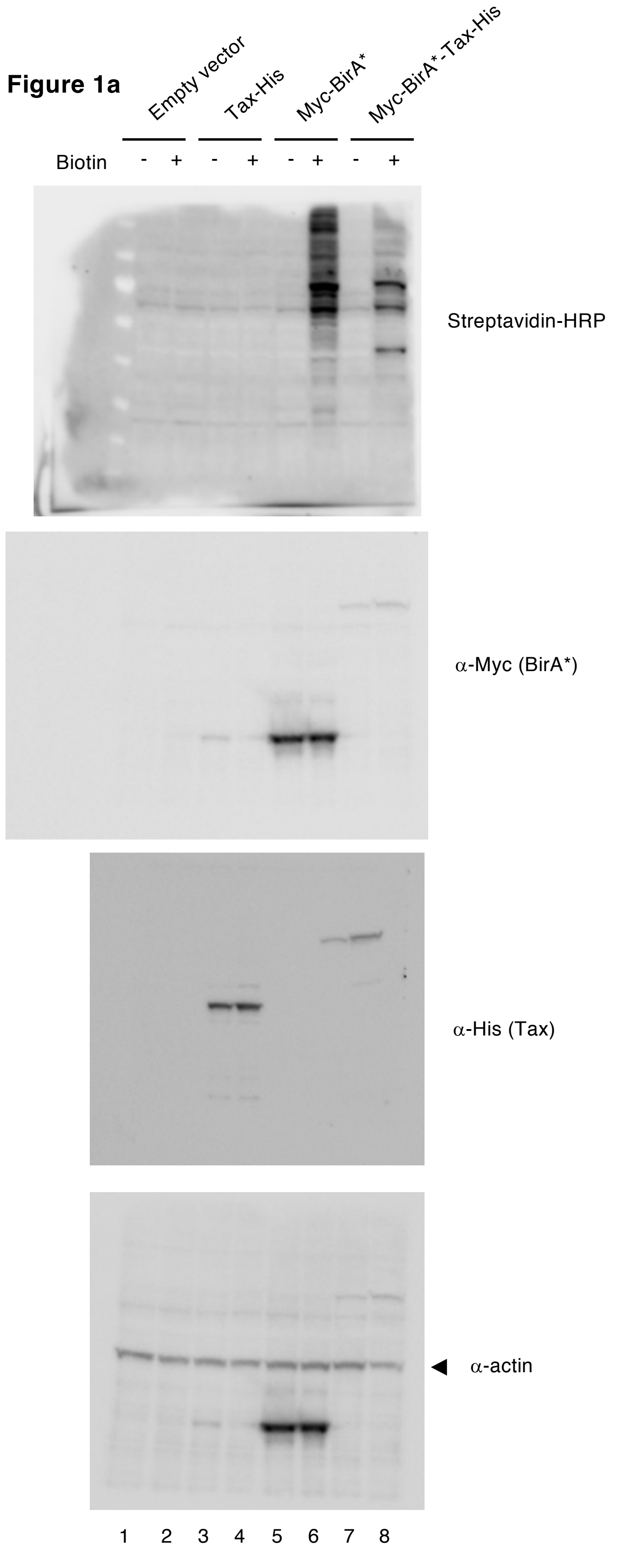

Figure 1d

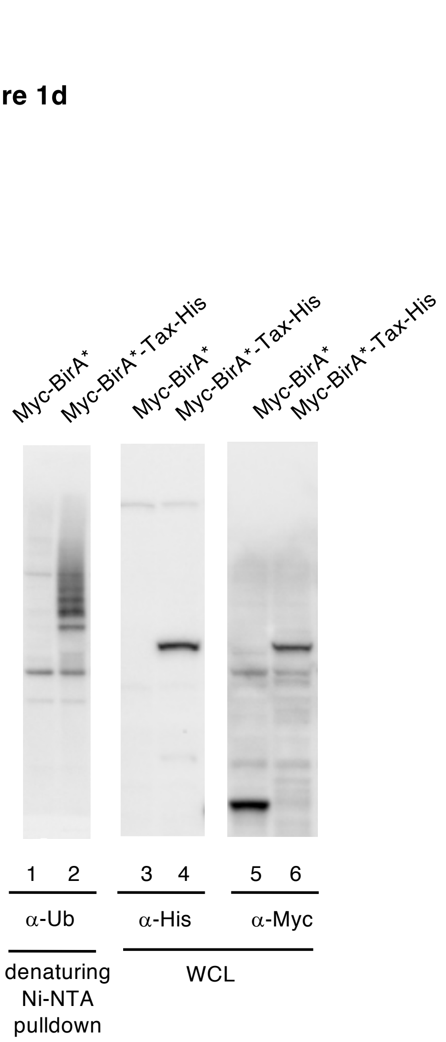

Supplementary Figure S4. Full-length blots and gels.

Figure 1f

JK : Jurkat cells, negative control
Hut: HuT102
C81: C8166
C91: C91PL

WCL (inputs)

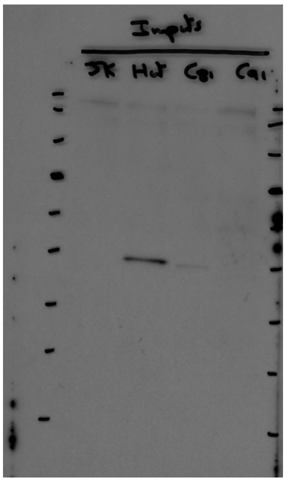

short exposure

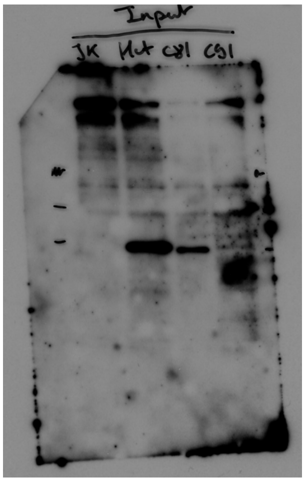

long exposure

◀ WB α-Tax

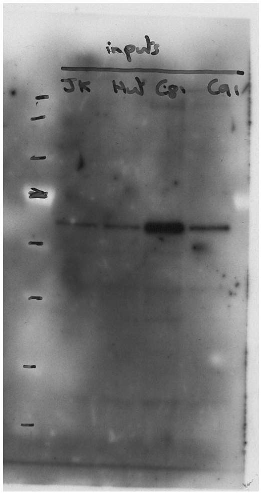

WB α-p62

IP α-p62 (IP) or control Ig (Ig)

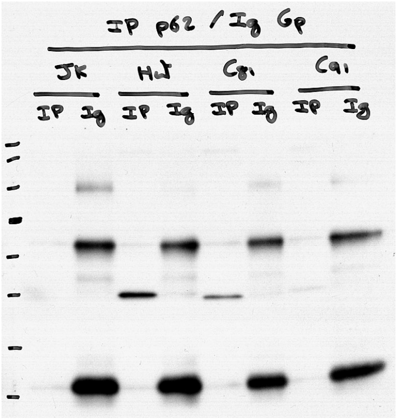

short exposure

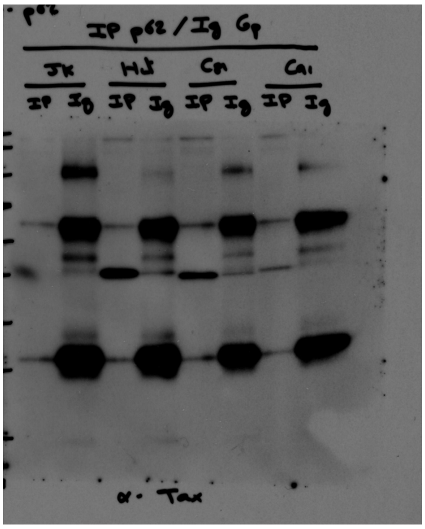

long exposure

◀ WB α-Tax

IP α-Tax or control Ig (Ig)

IP α-Tax
IP Ig

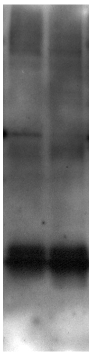

◀ α-p62

Supplementary Figure S4. Full-length blots and gels. (continued)

Figure 2c

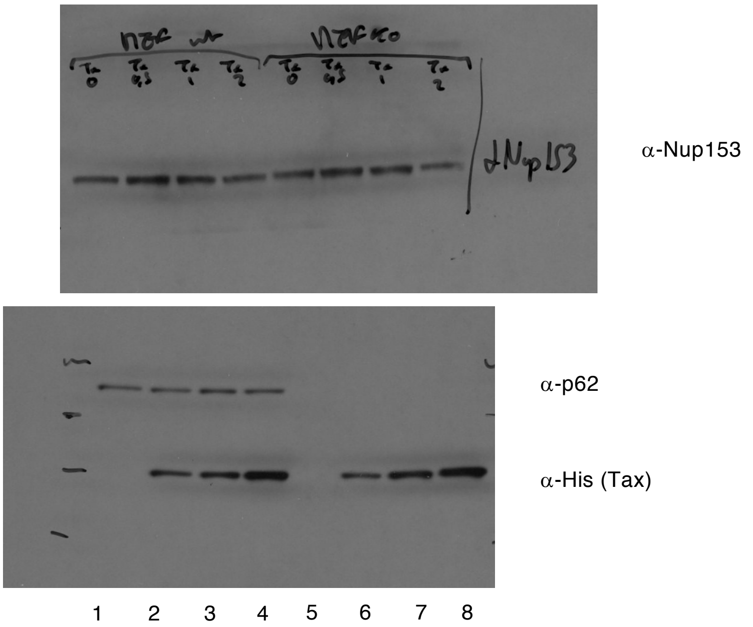

Figure 2d

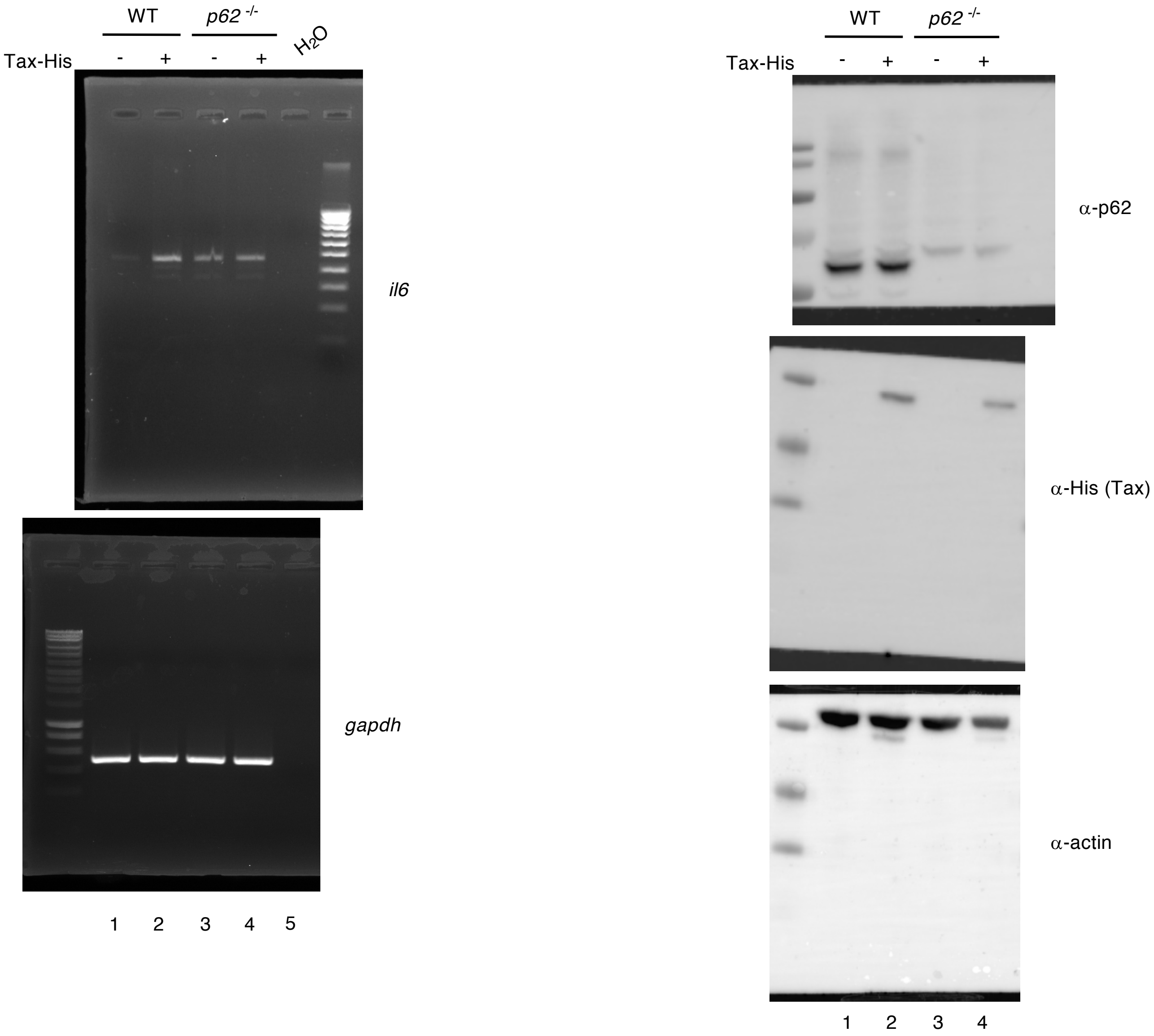

Supplementary Figure S4. Full-length blots and gels. (continued)

Figure 2f

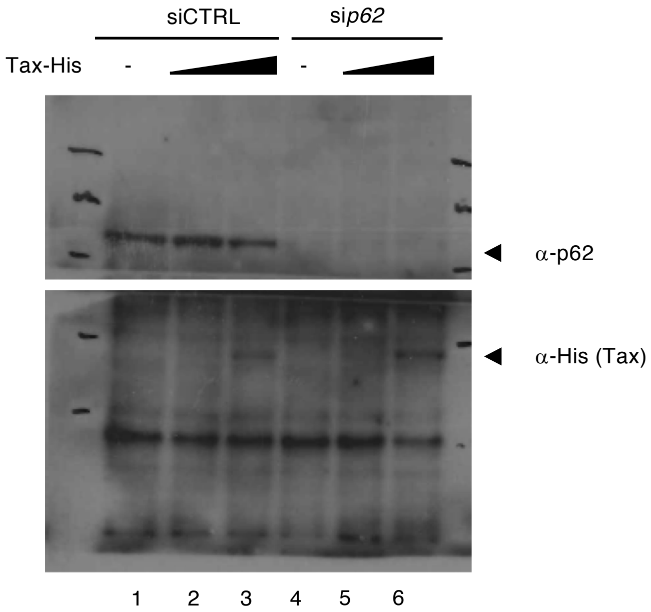

Supplementary Figure S4. Full-length blots and gels. (continued)

Figure 2h

G: Empty-IRES-GFP lentivector
T: Flag-Tax-IRES-GFP lentivector

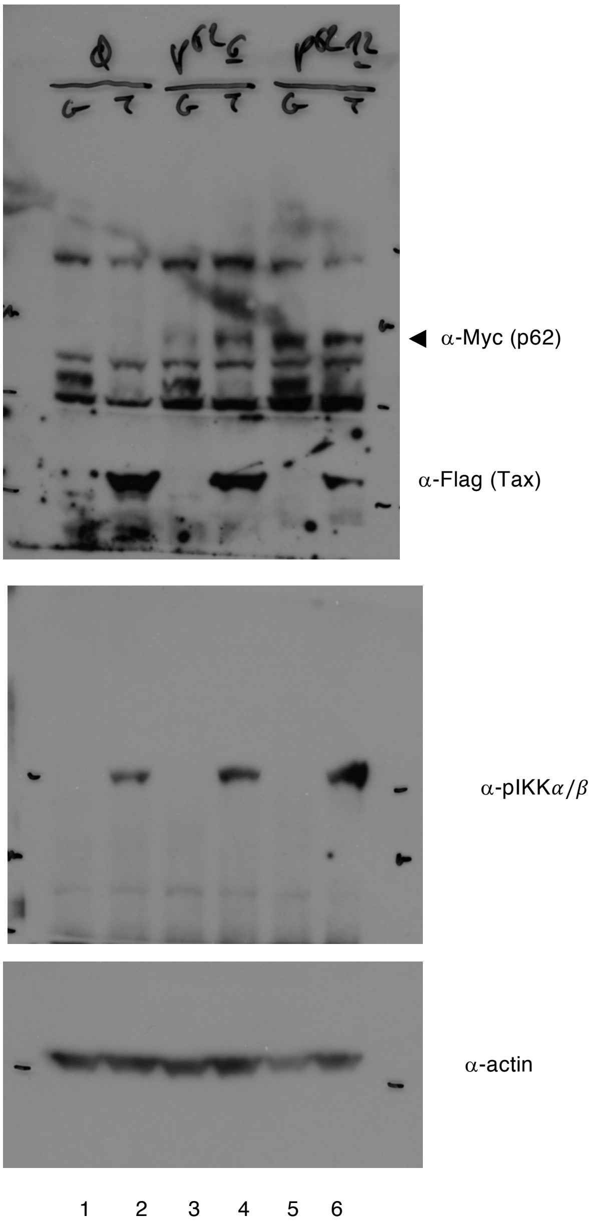

Supplementary Figure S4. Full-length blots and gels. (continued)

Figure 3c

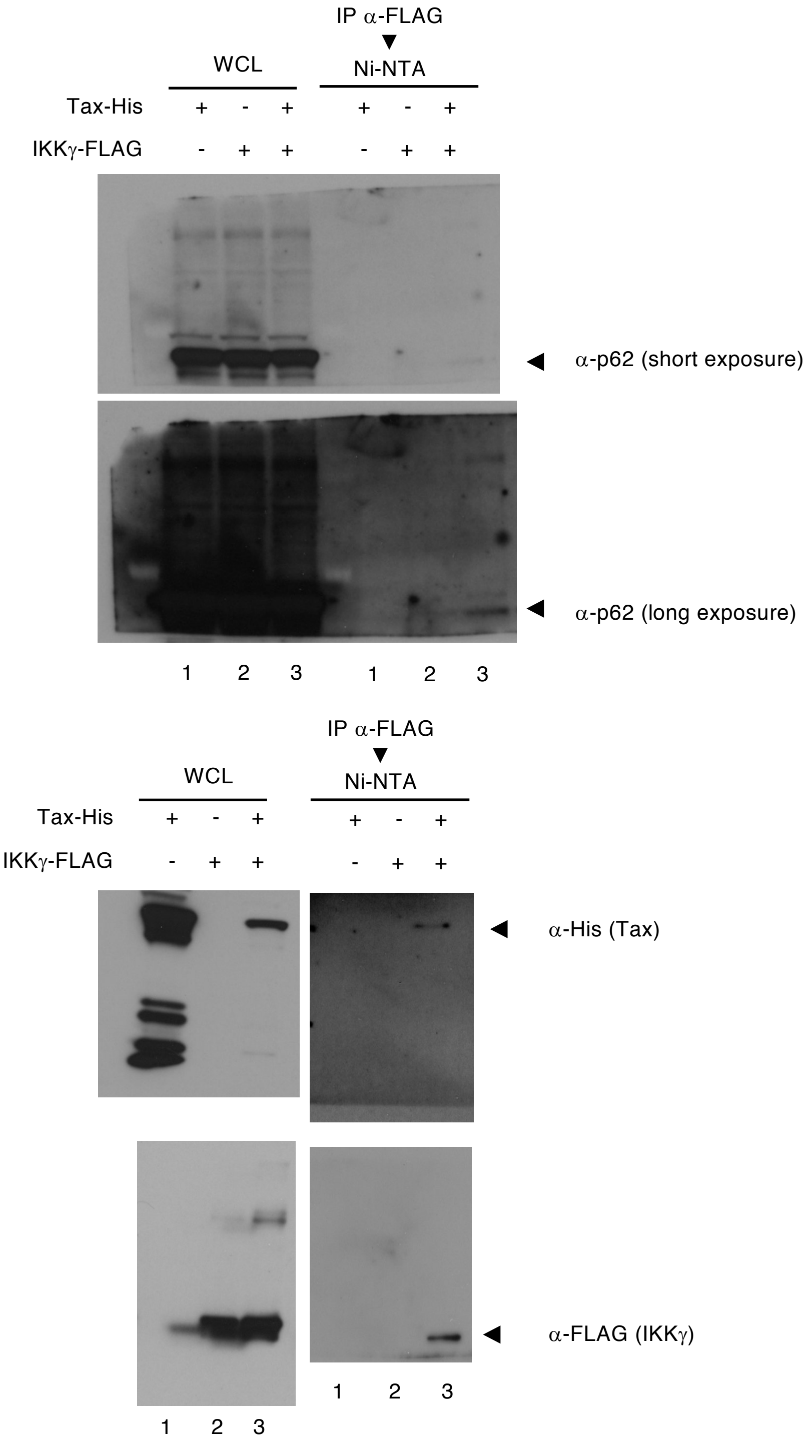

Supplementary Figure S4. Full-length blots and gels. (continued)

Figure 4b

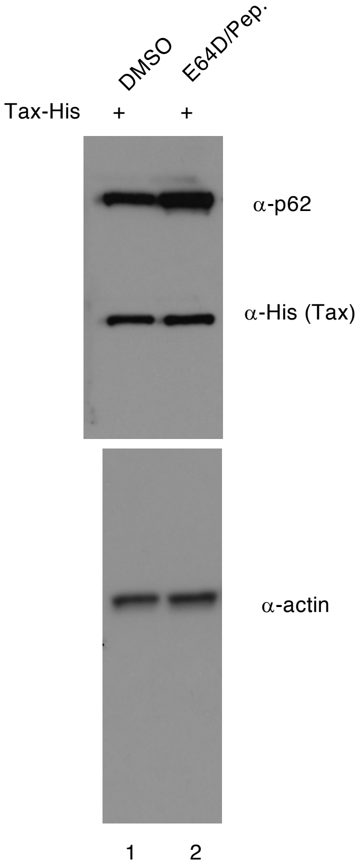

Figure 4c

D: DMEM
H: HBSS

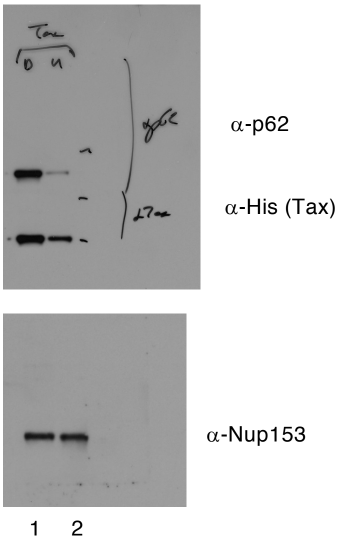

Supplementary Figure S4. Full-length blots and gels. (continued)

Figure 6b

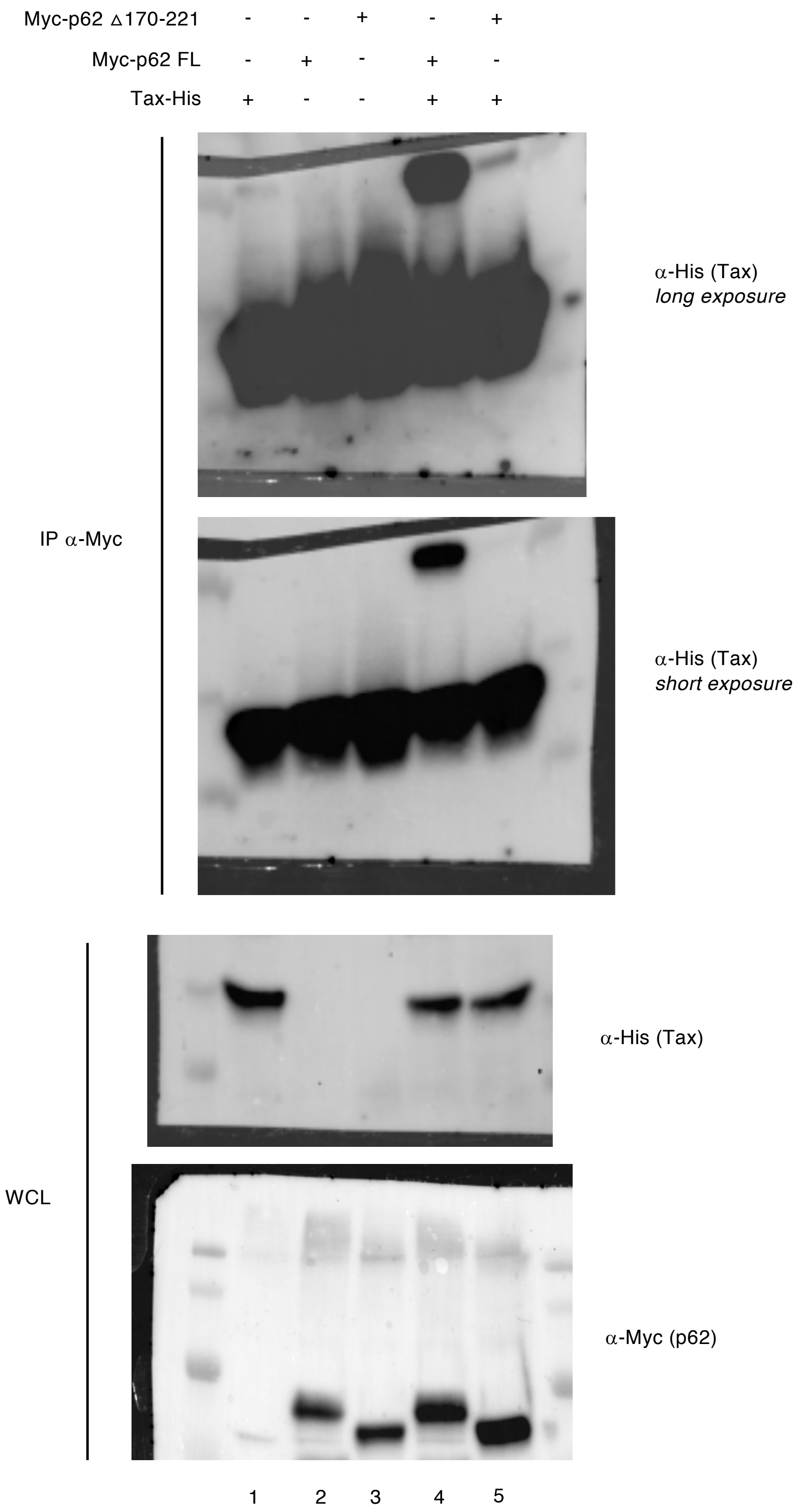

Supplementary Figure S4. Full-length blots and gels. (continued)

Figure 6c

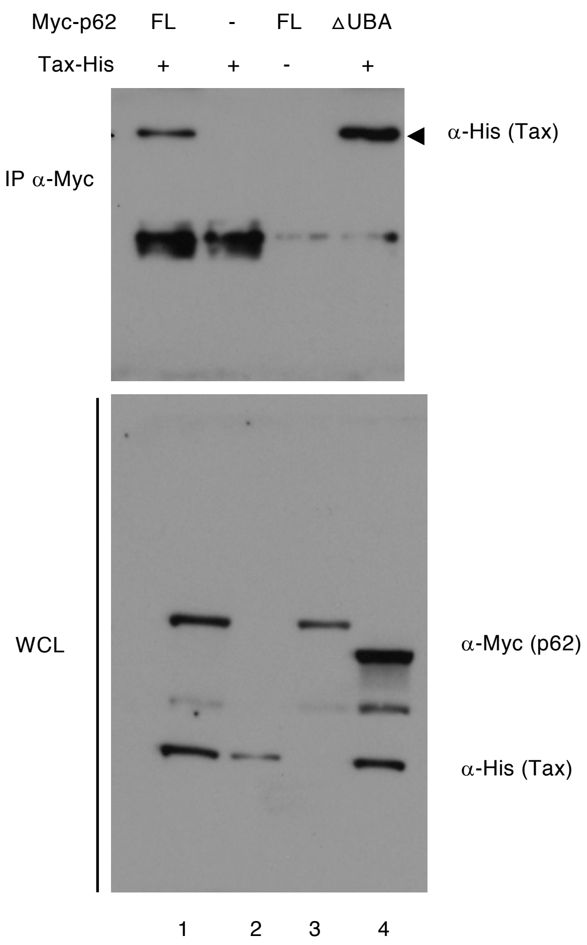

Figure 6d

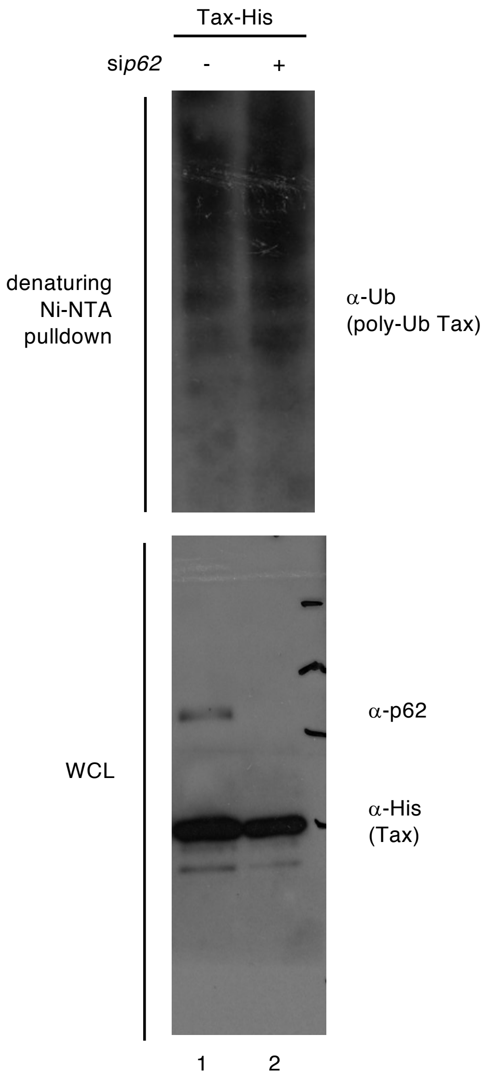

Supplementary Figure S4. Full-length blots and gels. (continued)

Figure 6e

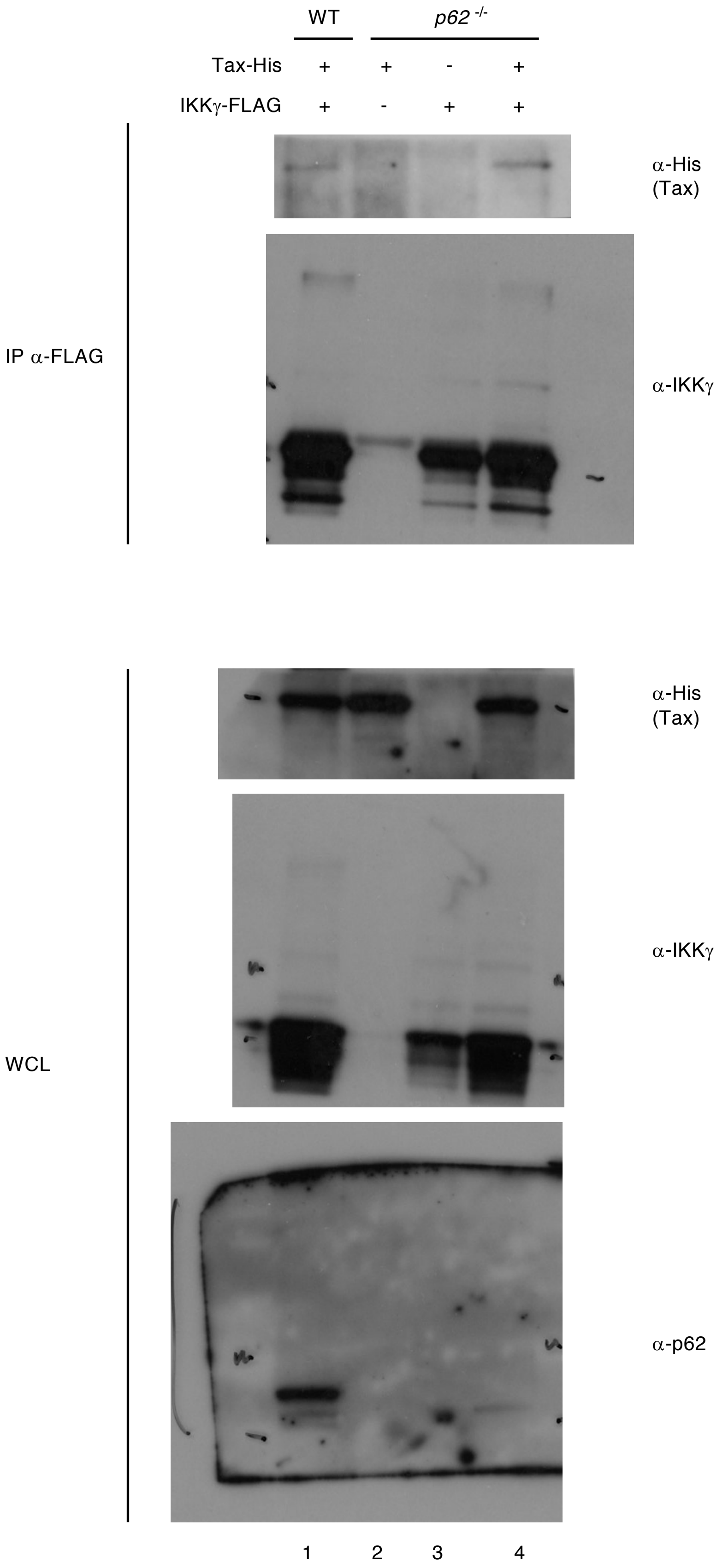
WT
p62 -/-
Tax-His + + - +
IKKγ-FLAG + - + +
IP α-FLAG
α-His
(Tax)
α-IKKγ
WCL
α-His
(Tax)
α-IKKγ
α-p62
1 2 3 4

Supp Figure S1b

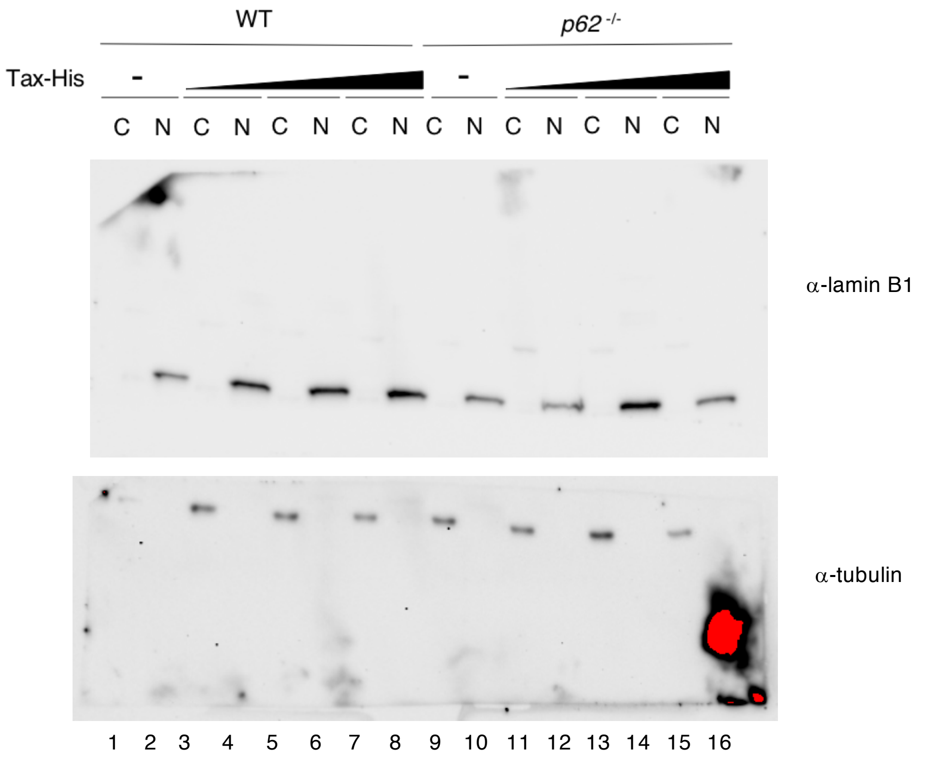

Supplementary Figure S4. Full-length blots and gels. (continued)

Supp Figure S1d

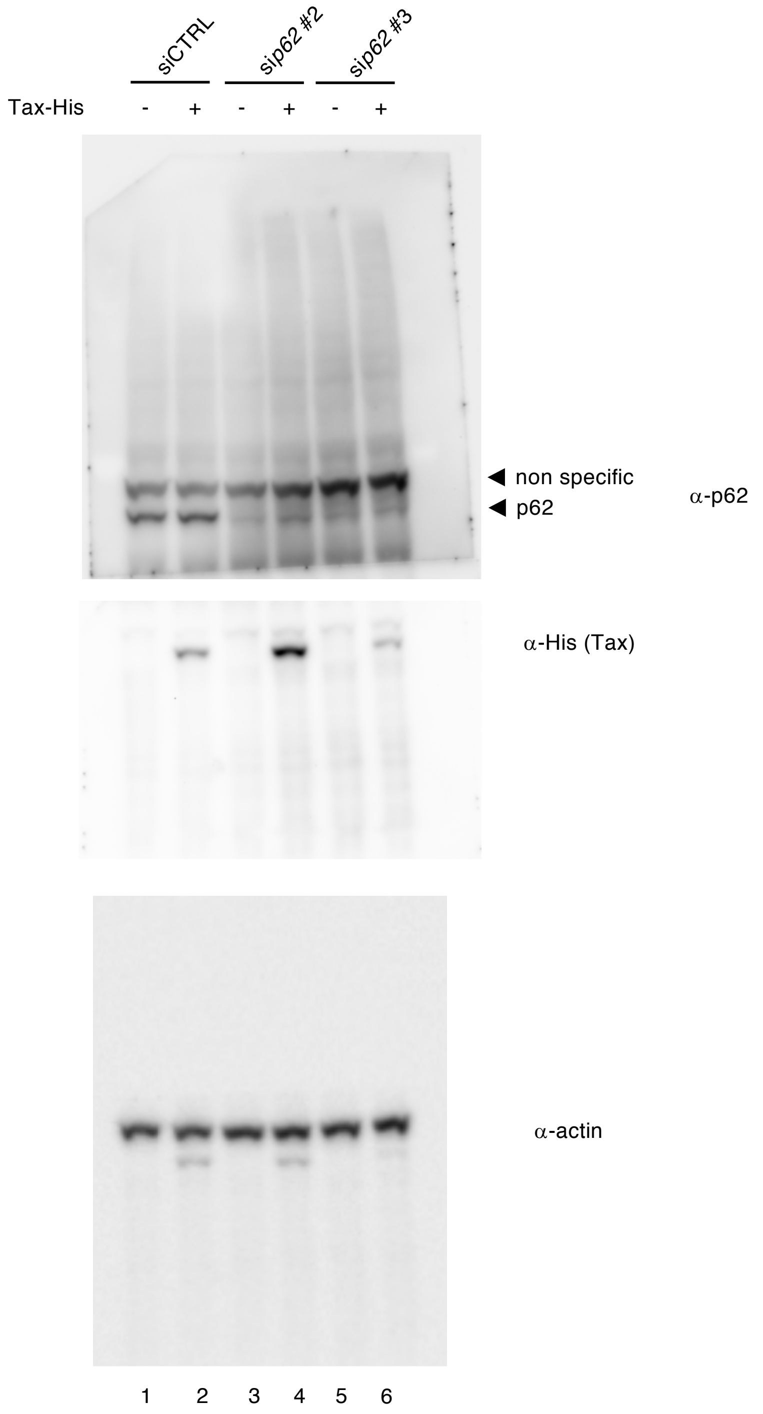

Supplementary Figure S4. Full-length blots and gels. (continued)

Supp Figure S1f

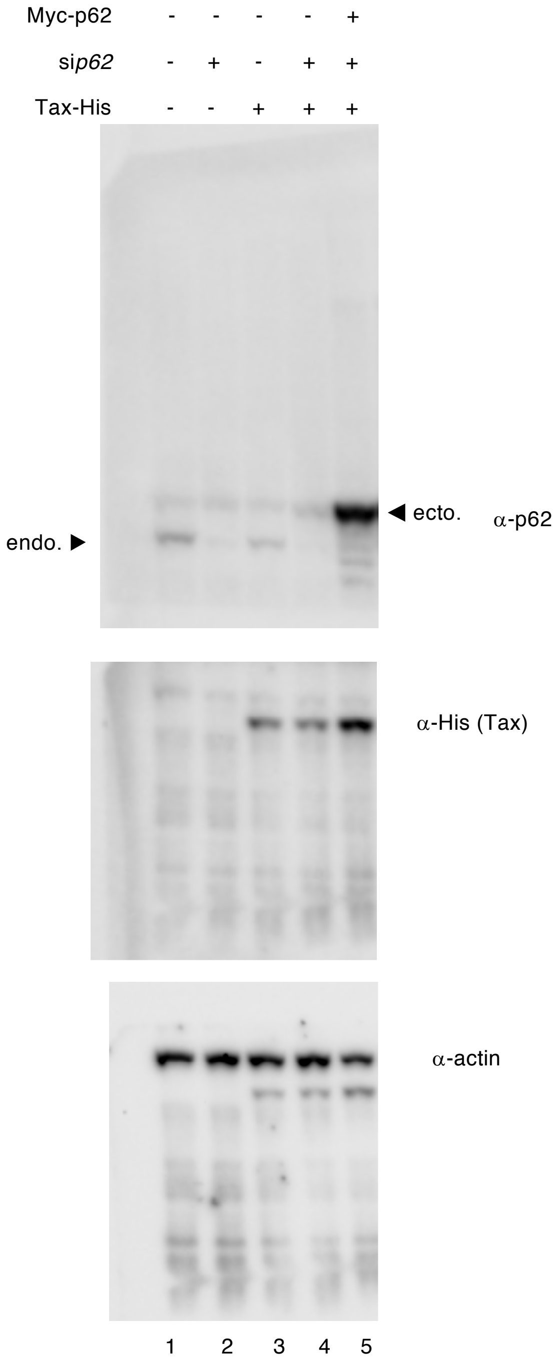

Supplementary Figure S4. Full-length blots and gels. (continued)

Supp Figure S1h

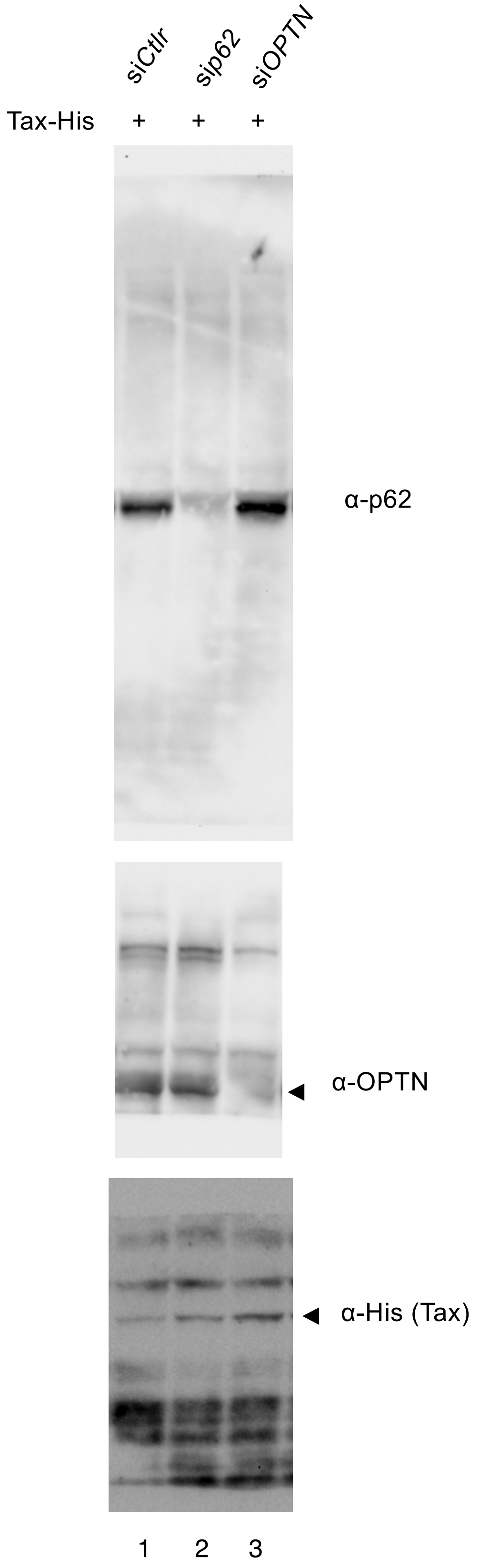

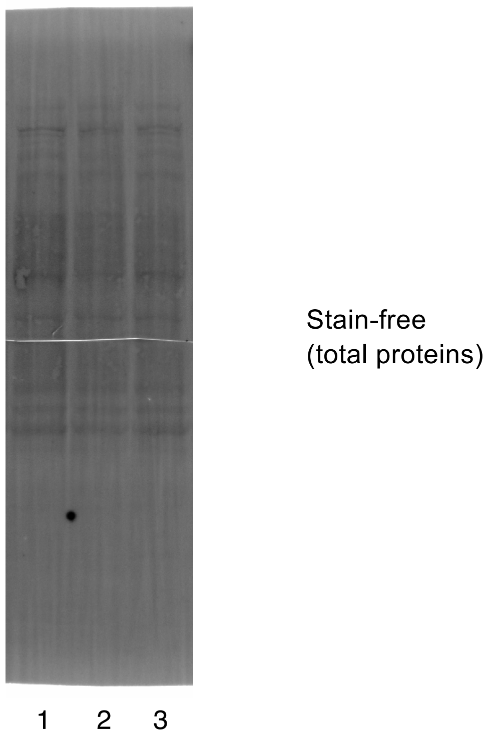

Supplementary Figure S4. Full-length blots and gels. (continued)

Supp Figure S2a

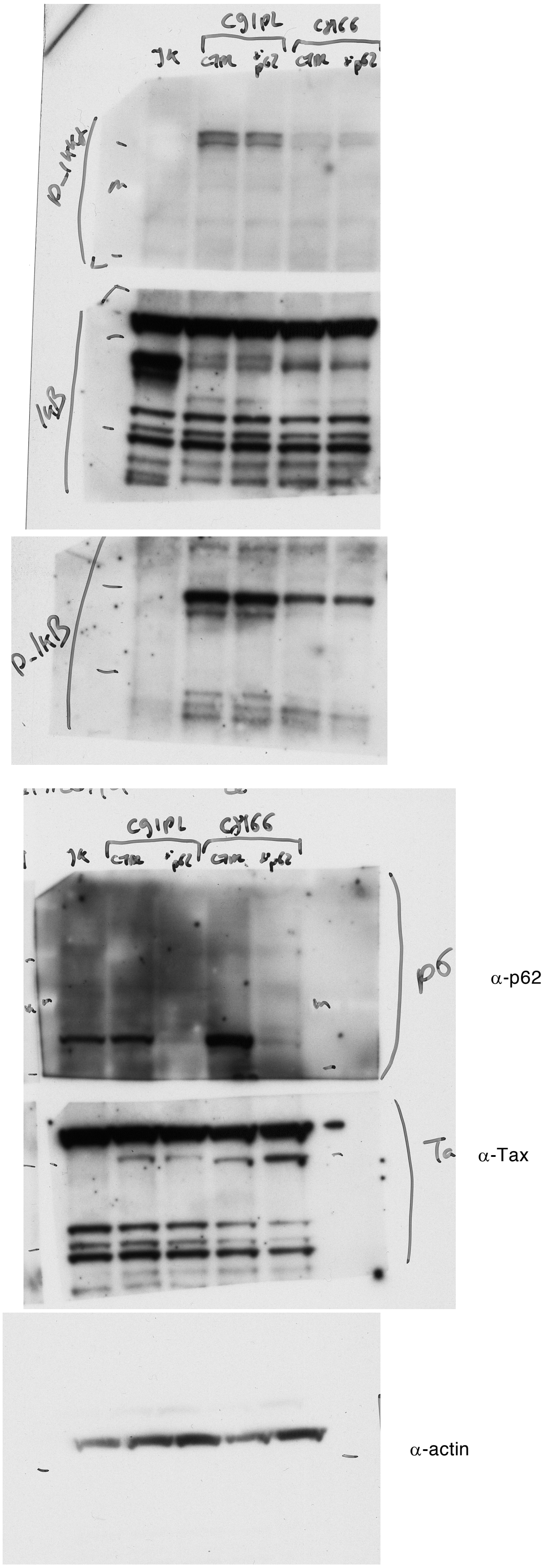

Supp Figure S2b

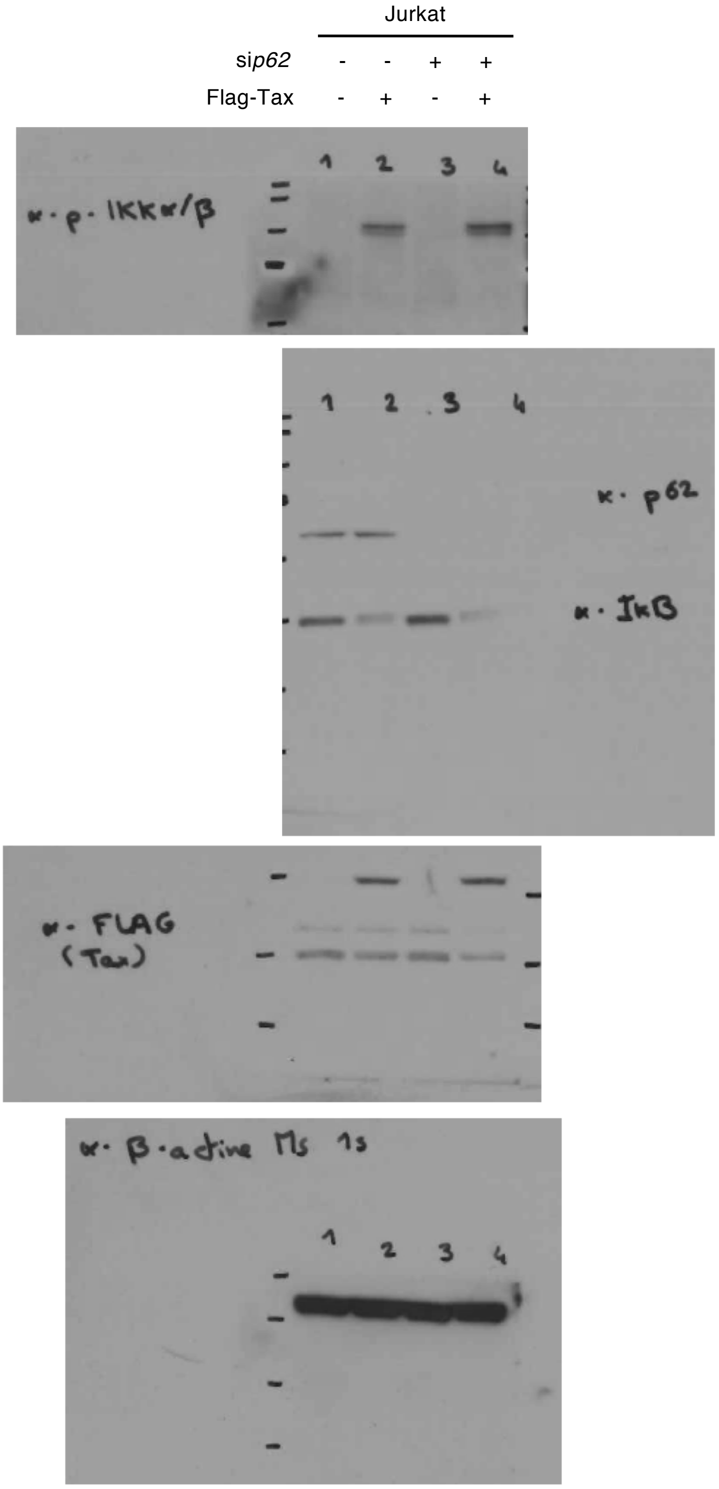

Supplementary Figure S4. Full-length blots and gels. (continued)

Supp Figure S3a

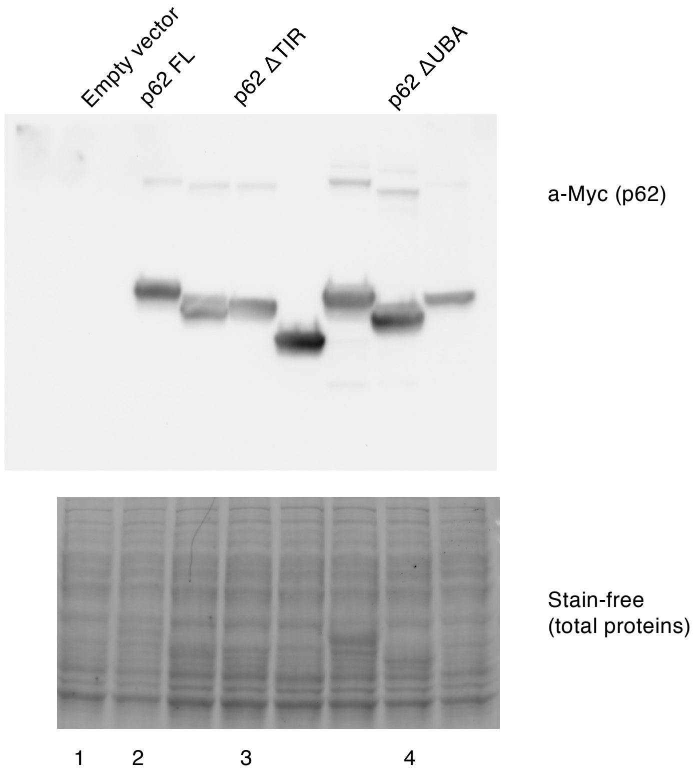

Supp Figure S3b

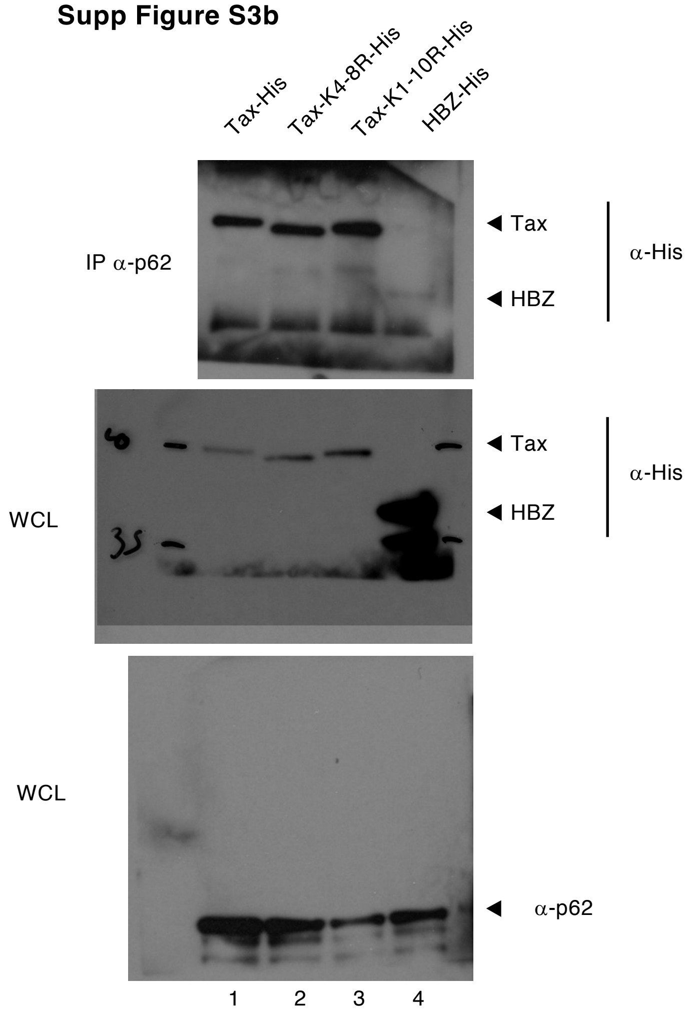

Supplementary Figure S4. Full-length blots and gels. (continued)
